# Assessing the effects of using high-quality data and high-resolution models in valuing flood protection services of mangroves

**DOI:** 10.1371/journal.pone.0220941

**Published:** 2019-08-20

**Authors:** Pelayo Menéndez, Iñigo J. Losada, Saúl Torres-Ortega, Alexandra Toimil, Michael W. Beck

**Affiliations:** 1 Environmental Hydraulics Institute (IHCantabria), Universidad de Cantabria—Avda, Isabel Torres, Parque Científico y Tecnológico de Cantabria, Santander, Spain; 2 Institute of Marine Sciences, University California Santa Cruz, Santa Cruz, CA, United States of America; KAUST University, SAUDI ARABIA

## Abstract

The rate of change on coastlines is accelerating from climate change and coastal development. Coastal flooding is a particularly pressing and increasing problem, which affects hundreds of millions of people and damages trillions of US$ in property. Scientists, practitioners and managers must be able to quickly assess flood risk and identify appropriate adaptation and risk reduction measures often with limited data and tools, particularly in developing countries. To inform these decision-making processes, we identify how sensitive flood risk and adaptation analyses are to changes in the resolution of data and models. We further do these comparisons in the context of assess the benefits of an ecosystem-based approach for risk reduction. There is growing interest in these ecosystem-based approaches as cost effective measures for adaptation and risk reduction. We assess flood risks from tropical cyclones and the flood risk reduction benefits provided by mangroves in Pagbilao (the Philippines). Then, we also compare risks and risk reduction (benefits) using different quality data and models, to identify where to invest in in new modeling and data acquisition to improve decision-making. We find that coastal flood risk valuation improves by using high resolution topography and long time series of data on tropical cyclones, while flood reduction benefits of mangroves are better valued by using consistent databases and models along the whole process rather than investing in single measures.

## Introduction

Coastal flooding effects are expected to enhance considerably during the 21^st^ century, due to three main reasons. First, development in the coastal zone, that has led to an increasing number of people and property located in coastal floodplains [1]. Second, the increase in intensity of extreme storms [2,3], such as recent tropical cyclones in 2017 (e.g., Franklin, Harvey, Irma, Katia, José and María), that will result in devastating consequences to people and property [4] and could accentuate with Sea Level Rise [5,6]. Third, the coastal ecosystem loss [7,8] reduces the protection capacity of coastal areas to climate hazards and increases flood risks.

As flood risks increase, there is a growing interest in the understanding of how natural ecosystems (e.g. coral reefs, mangroves and salt marshes), damp waves and reduce flood levels [9]. This is an important service to coastal communities and should be valued to inform policies for sustainable development, disaster risk reduction and environmental conservation. Mangroves are particularly relevant for risk reduction for many tropical nations [10–12], but they are being lost at an alarming rate. Given the increase in coastal risks, decision-makers must respond quickly, and they often have limited information, particularly for ecosystem-based solutions, that lead them to make wrong decisions, such as relying on traditional measures rather than prioritizing over green alternatives, even when data clearly show their limitations in effectiveness and cost [13,14].

Assessing flood risks requires key data, numerical models and statistical tools to assess flooding consequences to people and property. Consequently, the access to high quality and time-space homogeneous data is a growing need for decision-makers and coastal communities to accurately assess risk and value adaptation measures. The available input data, numerical models and statistical tools will likely decide the geographical scale at which any flood risk assessment analysis could be addressed (global, national, regional or local) [15]. While global (national and regional included) approaches are best suited for screening assessments identifying hotspots and supporting first national ranking of ecosystem services, local studies are appropriate for specific service valuation, risk reduction or adaptation projects implementation and cost-benefit analysis. Unfortunately, many local decisions are based on low accurate methods and low resolution data [16]. Most assessments of the sensitivity of flood risk models focus on exploring the sensitivity of coastal flood risk against single elements, such as the resolution of Digital Elevation Model (DEM) [17,18] and assets (i.e. spatial distribution of people and property [19]). No studies have explored the sensitivity of flood models for assessing risk reduction benefits of ecosystem-based adaptation measures, flood risks in the presence of coastal ecosystems and to other combined data and methods [20].

Comparing flood protection service value calculated using different approaches and datasets thus helps to quantify the order of magnitude of errors behind making direct use of simplified approaches or low-resolution datasets for local applications when there is a lack of local specific datasets and economic or technical resources. The aim of this study is to provide guidance on where to invest in new modeling and data acquisition to improve assessments of flood risk and ecosystem-based adaptation measures. To answer this question, we carry out a sensitivity analysis of flood risks to variations in the number of tropical cyclones, coastal segmentation, DEM resolution, flood methods and population data resolution. Each single element is individually tested and compared with the case of fully availability of high-resolution data and process-based models (Benchmark case).

## Methods and study site

### Methodology overview

**Fig 1:**
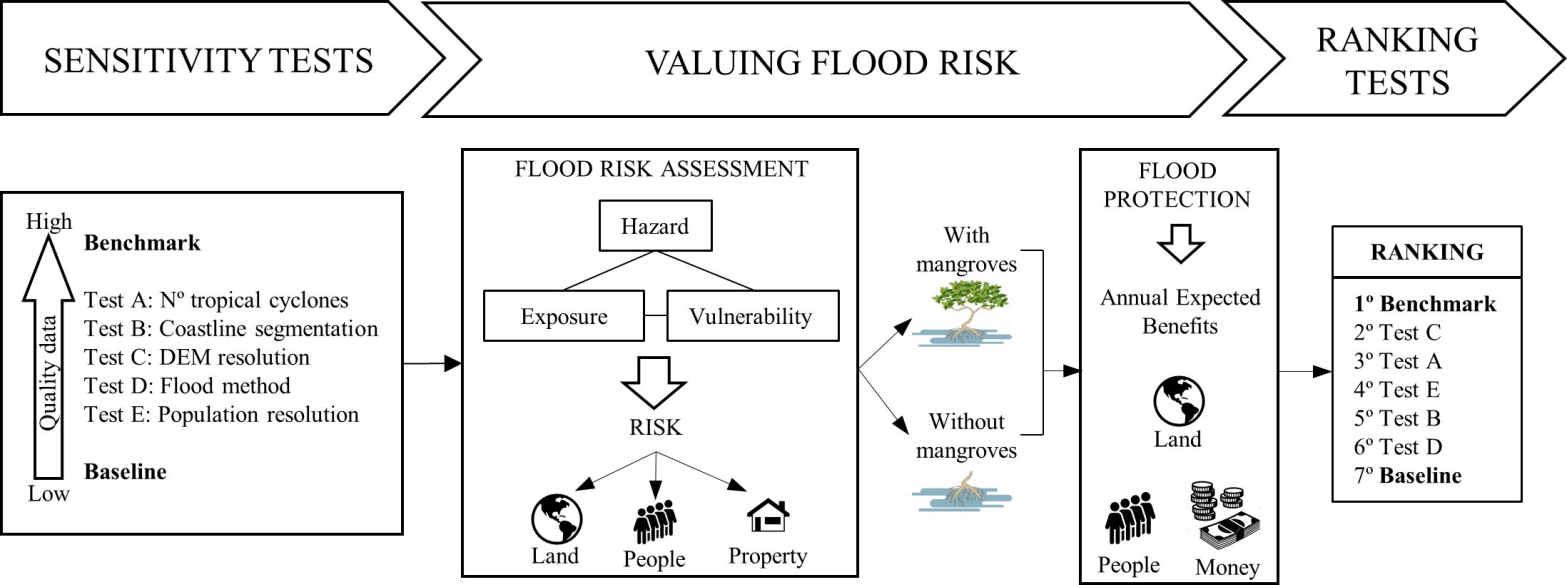
Workflow diagram and results. Strategy for testing the sensitivity of flood risk and flood protection benefits of mangroves to different approaches, with the aim of informing decision-makers where the maximum benefit is gained with improving data and models.

The workflow diagram (Fig 1) summarizes the process followed in this work to assess the sensitivity of flood risk and flood risk reductions to different sets of data and modeling tools.

To quantify risks, we follow a four-step methodology based on the risk assessment and management framework of the IPCC (Intergovernmental Panel on Climate Change) [5]: Hazard analysis includes steps 1 and 2; Impacts are calculated in step 3; and Exposure, Vulnerability and Risk are all evaluated in step 4. This multi-step methodology has been applied by others [10,11,21] and the output of each step is briefly described below:

1. Pre-habitat modeling: Offshore waves and storm surge statistical distributions produced by tropical cyclones propagated to the habitat.
2. Habitat modeling: The Total Water Level along the coast, namely Flood Height (FH), due to waves and surge propagation through mangrove fields.
3. Flood impacts: coastal flooding and local water depth.
4. Exposure, vulnerability and risk of flooding to land (km^2^), people (nº) and property (US$ millions of industrial and residential stock).

In this study we assess flood risk to annual expected floods for land, people and property, with and without mangroves. We also assess annual expected flood reduction benefits of adaptation measures to land, people and property.

Then we evaluate sensitivity of estimates of flood risk and adaptation benefits to variations in five key data and modeling elements (storms, coastline segmentation, topography, flood methods and exposure data). We compare each individual variation (sensitivity test) and the lowest resolution set for the five elements (Baseline case), with the highest resolution set (Benchmark case). In the sensitivity tests, we keep everything at low-resolution except for one, the variable where we higher the resolution.

#### Sensitivity tests

We ran sensitivity tests on all five variables (data and models). (a) *Storms* (*Number of tropical cyclones)*: Improving tropical cyclone´s historical databases by using larger time series (S1 Fig, S2 Fig and S3 Fig). (b) *Coastline segmentation (number of coastal cross-shore profiles)*: Increasing the number of coastal segments (S4 Fig). (c) *Topography (DEM resolution)*: Measuring the effect of improving the DEM horizontal resolution (S5 Fig). (d) *Flood method (Bathtub vs process-based)*: Comparing a stationary flood method based on hydraulic connectivity (bathtub method) with a process-based model (S6 Fig). (e) *Exposure data (Population resolution)*: Calculating the effect of using high resolution people distribution data versus coarse gridded population data (S7 Fig).

On the one hand, we use as our **Benchmark case** a high-resolution assessment of flood risk for Pagbilao, simultaneously using the best set of databases and models from the five sensitivity test, to provide site-specific results, that could, for example, be used for local service valuation, adaptation projects implementation and cost-benefits analysis. On the other hand, we consider as our **Baseline case** the currently available global data and model of coastal flood risk, simultaneously using the low-resolution set of the five sensitivity tests, to provide global or large regional risk assessment.

We calculate the Error Rate Index (ERI) (Eq 1) to compare Benchmark case (E_Benchmark_) with the Baseline case and each sensitivity test (E_i_). Sensitivity tests could either overestimate (ERI >0) or underestimate (ERI<0) Benchmark values. It allows us to compare sensitivities to data and models, with the aim of choosing the most efficient way to improve flood risk assessments from any global, national or regional scale.

$$ERI=100\cdot \left(E_{i}‐E_{Benchmark}\right)/E_{Benchmark}$$ (1)

Data and tools applied at each step of the methodology and for both, Baseline and Benchmark cases, are summarized in Table 1.

**Table 1:** Multi-step methodology to evaluate flood risks and flood reduction benefits of mangroves.

			BASELINE CASE	BENCHMARK CASE
**(1)**	**PRE-HABITAT MODELING**	**Data**	*Tropical cyclones: Historical IBTrACS [22] Astronomical Tide (GOT) Mean Sea Level Bathymetry global: GEBCO [23]	*Tropical cyclones: Synthetic [24] Astronomical Tide (GOT) Mean Sea Level Bathymetry global: GEBCO [23]
		**Tools**	Clustering method: DMA [25] Offshore: Delft3D model (2D mesh at 5 km) Nearshore: Delft3D model (2D mesh at 100 m)	Clustering method: DMA [25] Offshore: Delft3D model (2D mesh at 5 km) Nearshore: Delft3D model (2D mesh at 100 m)
**(2)**	**HABITAT MODELING**	**Data**	Bathymetry global: GEBCO [23] Bathymetry reefs: SeaWiFS [26] Mangroves 2010: WCMC [7] Coral Reefs: UNEP-WCMC	Bathymetry global: GEBCO [23] Bathymetry reefs: SeaWiFS [26] Mangroves 2010: DENR [27] Coral Reefs: UNEP-WCMC
		**Tools**	*Cross-shore Profile tracer (2 km) Delft3D model (1D mesh at 10m)	*Cross-shore Profile tracer (200 m) Delft3D model (1D mesh at 10m)
**(3)**	**FLOODING IMPACTS**	**Data**	*Topography: MERIT at 90 m [28] Coastline: GSHH [29]	*Topography: IFSAR at 5 m [30] Coastline: GSHH [29]
		**Tools**	*Flood method: Bathtub [21] Reconstruction method: RBF [31] Extreme distribution: Pareto Poisson	*Flood method: RFSM-EDA model [32] Reconstruction method: RBF [31] Extreme distribution: Pareto Poisson
**(4)**	**EXPOSURE, VULNERABILITY AND RISK**	**Data**	*Population data: GPW at 1 km [33] Property data: GAR15 [34] Damage functions: HAZUS [35]	*Population data: WorldPop at 100 m Property data: GAR15 [34] Damage functions: HAZUS [35]
		**Tools**	Downscaling people: from 1 km to 90 m Downscaling property: from 5 km to 90 m Annual Expected Function	Downscaling people: from 100 m to 5 m Downscaling property: from 5 km to 5 m Annual Expected Function

**Key data and tools for assessing flood risk and risk reduction benefits.** We show all the data and tools for the Benchmark and Baseline cases. We note with an asterisk (*) the variables that we assessed in sensitivity tests.

### Study area

We assess the coastal flood protection service provided by mangroves in Pagbilao (the Philippines), a municipality located in the southern part of Quezon Province in Luzon Island, the north coastline of Tabayas Bay. This study site was chosen because: first, its reasonable coastline extension for local high resolution analysis (~20 km); second, the availability of high-quality local data; third, the remarkable presence of mangroves [36], and fourth, its exposure to local extreme storms.

**Fig 2:**
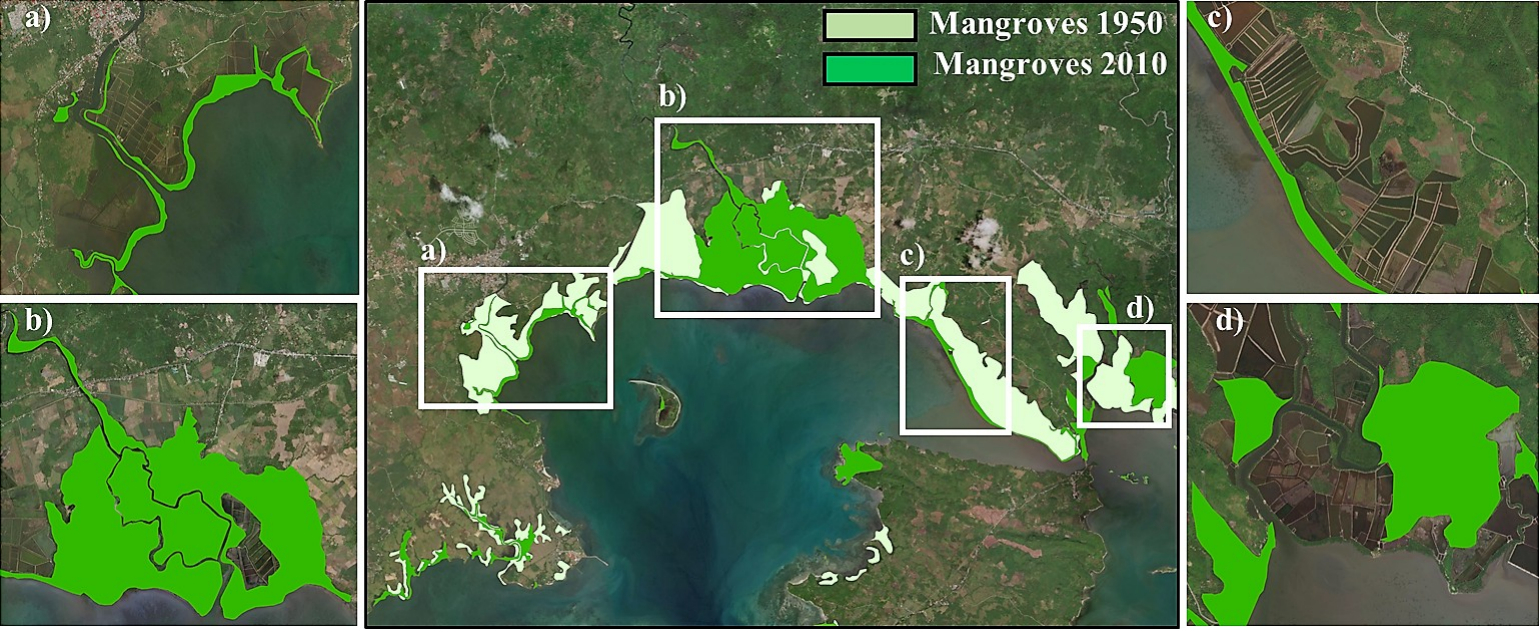
Mangrove cover in Pagbilao (The Philippines). Mangroves extent in 1950 and 2010 in Pagbilao municipality, zooming in four different areas with different mangrove covert development: (a) Mangroves retreat may be due to a change in the river sediment transport in the west side of the municipality, (b) mangroves density increment due to restoration policies, (c) mangroves conversion into aquaculture areas and (d) mangroves migration in the east side of Pagbilao. Reprinted from ArcGIS Online maps under a CC BY license, with permission from Esri, original Copyright 2018 Esri (Basemaps supported by Esri, DigitalGlobe, GeoEye, Earthstar Geographics, CNES/Airbus Ds, USDA, AEX, Getmapping, Aerogrid, IGN, IGP, swisstopo, and the GIS User Community).

Pagbilao covers 15,820 ha, whereof 4,560 ha are mangrove forests. It has a total population of 75,000 people of which 27,958 live in low-lying areas exposed to flood threats, and it accounts for US$45.06 million of property located in potentially flooded areas (US$16.28 million of industrial stock and US$28.78 million of residential stock). There has been a 43% decline in mangroves since 1950 mainly because of aquaculture, which has reduced mangroves to a narrow coastal band only hundreds of meters wide (Fig 2). The observed habitat decline over the last decades built a real concern in the conservation of natural resources [37] and management alternatives for the Pagbilao mangroves [38].

Coastal flooding in Pagbilao climate is mainly caused by tropical cyclones. The maximum observed surge in the offshore area of Pagbilao bay does not exceed 2 m and is produced by storms with a southeast-northwest track when crossing it.

**Fig 3:**
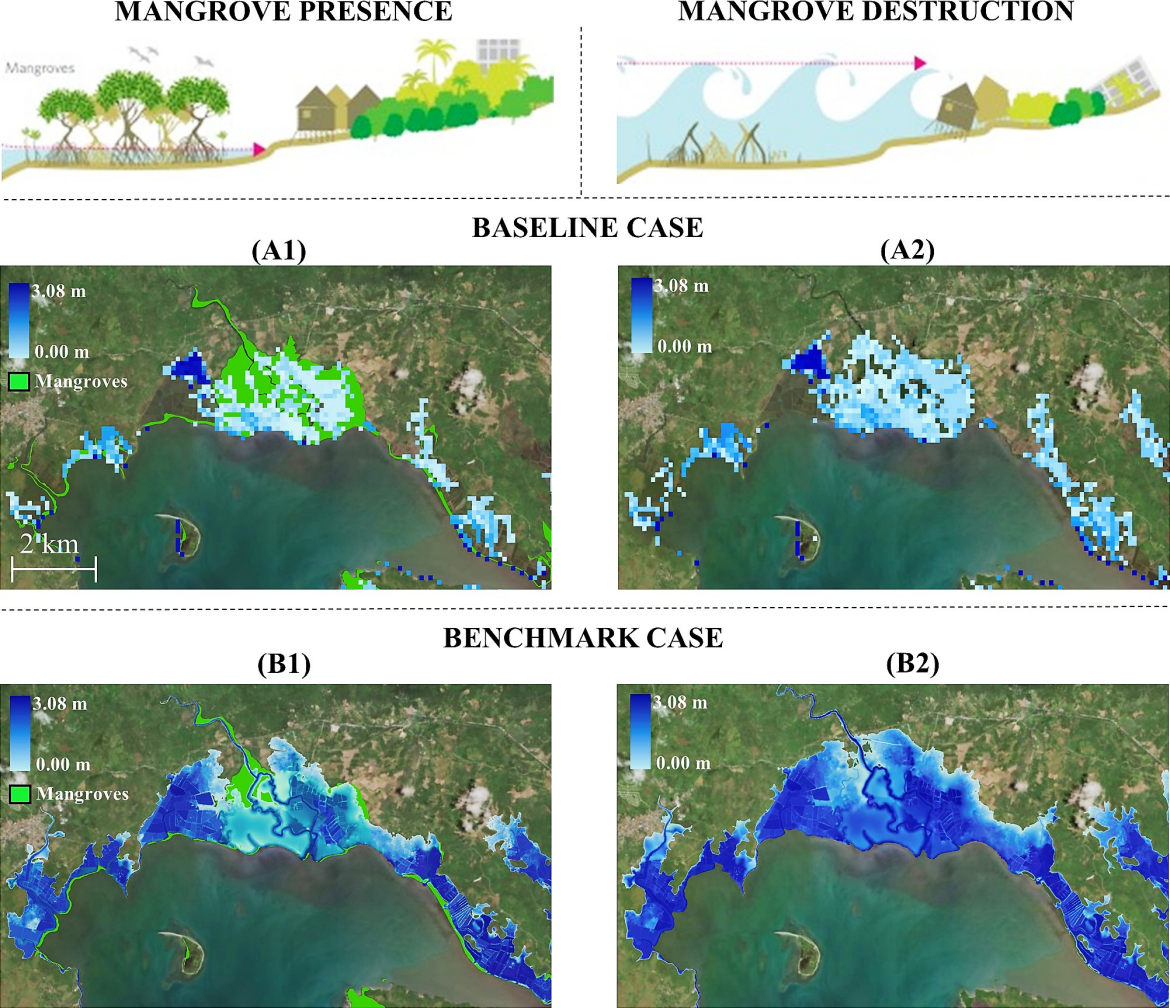
Flood map comparison in Pagbilao. Coastal flooding produced by 1-in-50 years tropical cyclone in Pagbilao. Comparison between Baseline case (with and without mangroves) and Benchmark case (with and without mangroves). Reprinted from ArcGIS Online maps under a CC BY license, with permission from Esri, original Copyright 2018 Esri (Basemaps supported by Esri, DigitalGlobe, GeoEye, Earthstar Geographics, CNES/Airbus Ds, USDA, AEX, Getmapping, Aerogrid, IGN, IGP, swisstopo, and the GIS User Community).

Significant differences are observed between Baseline (Fig 3A1 and 3A2) and Benchmark (Fig 3B1 and 3B2) cases, which illustrates the land area flooded by a 1-in-50-year storm with (Fig 3A1 and 3B1) and without (Fig 3A2 and 3B2) mangroves in Pagbilao Bay. In the Baseline case the flooding surface is underestimated (upper charts), while the Benchmark case significantly improves the quality (more pixel resolution) resulting in a larger flooded area.

## Results

We assessed the sensitivity of each of the variables individually and in combination for assessing flood risk and adaptation measures (Table 2 and S8 Fig). In terms of risk, increasing DEM resolution (Test “c”) always overestimates land, people and property flooded. Meanwhile, using higher resolution flood models (Test “d”) leads to significant underestimations of flood risk (when coupled with low resolution data). In terms of risk reduction, we find that benefits to people and property are less sensitive than risks to single improvements in data and models. It highlights the importance of being consistent in databases resolution along the whole methodology (e.g. applying Baseline case) rather than investing in individual high-resolution databases or process-based models.

**Table 2:** Flood risk and adaptation benefits assessment.

	LAND FLOODED (Hectares)			PEOPLE AFFECTED (Nº)			PROPERTY DAMAGE (US$ mill)		
	With	Without	Benefit	With	Without	Benefit	With	Without	Benefit
**Benchmark case (high-resolution)**	**320**	**350**	**30**	**3898**	**4510**	**612**	**4.23**	**5.05**	**0.82**
**Baseline case (low-resolution)**	**139**	**216**	**77**	**696**	**1268**	**572**	**1.22**	**1.94**	**0.72**
Test A: Nº of storms	182	256	74	1053	1701	648	1.68	2.43	0.75
Test B: Nº of profiles	137	214	77	683	1271	588	1.20	1.95	0.75
Test C: DEM resolution	340	380	40	5954	6653	699	7.80	8.95	1.15
Test D: Flood method	21	35	14	299	523	224	0.57	0.82	0.25
Test E: Exposure resolution (Pop)	139	216	77	736	1323	587	1.22	1.94	0.72

Flood risk (with and without mangroves) and risk reduction benefits to land, people and property. The benefits provided by mangroves are the difference in flooding with and without mangroves.

We use the values given in Table 2 to calculate the error index, ERI (Eq.1), with the aim of assessing sensitivity that would most help improve risk assessments. Higher absolute ERI’s indicate greater errors and lower sensitivity. The first ranking table (Table 3) sorts each case according to land, people and property risk estimates. S1 Table is similar but expressed in terms of assessment of adaptation options. The DEM resolution is, by itself, the most efficient way of reducing errors to flood risk to land and people. However, improving the DEM had relatively high errors for assessing impacts of flooding to property. Overall, as it is shown in Table 3, using the Baseline case is not always the worst option, which highlights that there are sensitivity tests, that, on their own, do not bring any improvement to the analysis and must be combined with other improvements so that it is worth investing resources and time in their use.

**Table 3:** Ranking table for valuing risks, based on ERI index.

RISK								
LAND			PEOPLE			PROPERTY		
**Rank**	**Sensitivity test**	**ERI**	**Rank**	**Sensitivity test**	**ERI**	**Rank**	**Sensitivity test**	**ERI**
1	DEM res.	+7.41%	1	DEM res.	+50.13%	1	Nº storms	-56.08%
2	Nº storms	-34.99%	2	Nº stoms	-67.63%	**2**	**Baseline**	**-66.37%**
**3**	**Baseline**	**-47.42%**	3	Exposure res. (Pop)	-75.89%	3	Exposure res. (Pop)	-66.37%
4	Exposure res. (Pop)	-47.42%	**4**	**Baseline**	**-77.01%**	4	Nº profiles	-66.51%
5	Nº profiles	-48.02%	5	Nº profiles	-77.15%	5	DEM res.	+80.81%
6	Flood method	-91.72%	6	Flood method	-90.37%	6	Flood method	-85.14%

A comparison of sensitivity of estimates of risk to 5 different factors and to the Baseline case. Baseline case in also ranked to show which elements improve risk estimates (ranked above the Baseline) and which do not (ranked below the Baseline). Risk is assessed as flooding of land (left), people (mid) and property (right).

We also assess the most efficient way of valuing risks in the presence of mangroves (S2 Table) versus in the absence of mangroves (S3 Table). We not only studied each single improvement of data and models individually, but also any possible combination of cases. S4 Table lists all the ERI indices of each existing combination, so that we can identify the most efficient way of combining datasets and modeling methods to reduce the error of flood risk estimates.

For a better understanding of the effect that each single improvement in datasets and methods has, we analyze, one by one, the sensitivity of flood risks to each case:

### Sensitivity to the number of storms (tropical cyclones)

Longer time series of storms (i.e. 1,000 years of synthetic tropical cyclones) results in better accuracy in flood predictions than shorter time series (i.e. 71 years of historical IBTrACS dataset) (S1 Fig and S2 Fig). For example, by increasing the number of tropical cyclones, risk assessment in presence of mangroves to land, people and property increases by 43 ha (+31%), 357 people (+51%) and US$ 0.46 million (+38%) with respect the Baseline case (calculated from Table 2). Additionally, we better estimate floods in no-mangrove scenarios (47% of average error) than in mangrove protected coastlines (59% of average error) if using synthetic tropical cyclones (S2 and S3 Tables).

### Sensitivity to the number of profiles

Coastal impacts (land flooded) and risks (people affected and property damaged due to coastal flooding) are slightly underestimated if moving from 2 km to a 200 m longshore segmentation. Table 2 shows that the area flooded, people affected and property loss in presence of mangroves decreases by 2 ha (-1.5%), 13 people (-1.8%) and US$ 0.02 million (-1.6%) with respect to the Baseline case.

### Sensitivity to DEM resolution

Overall predictions of flood risk to land and people was the most sensitive to changes in DEM. In presence of mangroves, coastal risk assessment increases by 201 ha (+144%), 5,258 people (+755%) and US$ 6.58 million (+539%) if using high resolution DEM (IFSAR 5m) instead of global DEM (MERIT 90 m). We observe in Table 3, that local high resolution DEM reduce errors with respect to the Baseline case when valuing land flooded (+7.41% vs -47.42%) and people affected (+50.13% vs -77.01%), but not property damages based on 5 km resolution dataset (80.81% vs -66.37%), due to the abrupt differences between the DEM and property data resolution (5 m and 5 km respectively).

### Sensitivity to flood method

The use of a process-based flood model (RFSM-EDA), instead of the bathtub method, results in lower estimates of risk with respect to the Baseline case: -118 hectares (-85%), -397 people (-57%) and US$ -0.65 million (-53%). These differences observed in Table 2 lead to an average error increment of 23% (Table 3) with respect to the Baseline case. However, if we combine the RFSM-EDA model with high resolution topography data (IFSAR 5 m), the error in risk assessment to land, people and property would be reduced to -36%, +40% and +67% respectively (S4 Table, row 14, column 1: “Flood”, column 2: “People” and column 3: “Property”).

### Sensitivity to population resolution

Using high-resolution population distribution (100 m WorldPop) as an alternative to low resolution data (1 km GWP) does improve the estimation of people affected by coastal flooding by 40 people (+6%), but not enough to reach the predicted 3,898 people by the Benchmark case (Table 2).

## Discussion

Given the growing risks of people and property to flooding and the need to quickly decide on effective risk reduction solutions, we identified the key factors that would most help improve upon existing global data or poor data sites and models for applications at national and potentially site-specific levels.

The assessment of flood risk to land and people is most sensitive to resolution of the topographic data. That is, you would benefit the most from making improvements in topography but also in the storm data (ranked #2 in both cases). Meanwhile, estimates of risks to property are most sensitive to the quality of the storms database. Consequently, improving DEM resolution is the most effective way of improving risk to land and people, but storms data may be the most important overall for improving risk estimates. We believe the results are particularly sensitive to storms data because, since we analyze damage in a statistical way (i.e. annual expected function), results are highly dependent on the number of elements considered. Increasing the number of tropical cyclones reduces uncertainty in the extreme value distribution analysis and improves overall annual expected risk estimates.

Conversely improvements of the flood method had the least improvement in all the risk assessments. That is, in many instances using a bathtub model is a fine approximation given what is available in the other databases.

In terms of assessing benefits from adaptation options, the model results were most sensitive to changes in the number of coastal segments to estimate people and property benefits, and changes in DEM and flood method to estimate land flooded benefits. These findings are different from the flood risk assessment because benefits are less sensitive than risks to database and model improvements and, consequently, to better estimate them, it is more important to use consistent databases and models along the whole process rather than investing in single measures.

However, it is sometimes effective to properly combine different elements to achieve the best results. For instance, using process-based flooding model (i.e. RFSM-EDA) underestimate flooding unless combined with high-resolution elevation data.

We expect these results to be broadly applicable to many areas but there are some particulars of the Pagbilao case that are important: (1) Pagbilao has experienced many cyclones. The effectiveness of increasing the number of tropical cyclones is more noticeable in coastal areas with high cyclone activity because it allows to generate a wider range of synthetic tropical cyclone intensities and tracks, improving the statistical analysis of extreme value distribution by narrowing confidence bands, especially for high return period storms (see S1 Fig and S2 Fig). (2) Pagbilao has a relatively simple and homogeneous coastline. That is why using 200 m spaced cross-shore profiles, rather than 2 km, do not significantly improve risk estimations in Pagbilao. This findings should not be projected to other regions with longshore morphology variability, different mangrove species and more complex distribution of coastal assets, which are probably more sensitive to the increase of coastal segmentation. (3) Pagbilao does not have available high-resolution socioeconomic data. The abrupt downscaling required to rescale the global 5 km grid data of property to 5 m (the same than high-resolution DEM) leads to an spatial misallocation of coastal exposure and the consequent overestimation of the capital loss. This fact reduces the efficiency of using high-resolution DEM. (4) Pagbilao is a local area with short coastline (~20km of coastline), where using process-based models (e.g. RFSM-EDA) are computationally affordable. However, it is no longer applicable spatial scales larger than 100 km because its time-consuming pre-processing mesh generation.

Further methodological limitations come up when valuing flood risk at different locations. First, calculating annual expected values of risk and benefits requires large sample data to reduce errors. However, historical datasets do not always provide enough information. Second, one-dimensional propagations neglect two-dimensional processes such as longshore currents or waves diffraction, missing some energy losses, that could be relevant in longshore varied coastal areas. Third, using 1 km bathymetry data is a high limitation to wave hydrodynamics modelling, especially in coral reef and mangroves environments due to the inability to capture the level of detail required to model coastal ecosystems processes. For that reason, we increase the accuracy in the vegetated shallow areas using both, local specific bathymetry of ecosystems [26] and parameterized profiles to correct bathymetric errors (S9 Fig).

In brief, for local high-resolution flood risk analysis, we must consider how to combine databases and methods to obtain the best possible result with the less effort and resources expenses, before making directly use of all the high-resolution databases available.

## Supporting information

S1 File Sensitivity tests description: S1_File_Sensitivity_tests_description. (DOCX)

S1 Fig Sensitivity Test A: Offshore H_S_ distribution in Tabayas Bay (Pagbilao). Offshore maximum significant wave height extreme distribution produced by (A) historical tropical cyclones and (B) synthetic tropical cyclones. Black circles represent the most probable value of H_S_. The solid line represents the best fit adjustment of the most probable values of H_S_. Dashed lines represent the 95% confidence interval of the analytical extreme value distribution. (TIF).
(TIF)

S2 Fig Sensitivity Test A: Offshore SS distribution in Tabayas Bay (Pagbilao). (A) Offshore maximum storm surge produced by historical tropical cyclones. (B) Offshore maximum storm surge produced by synthetic tropical cyclones. Black circles represent the most probable value of SS. The solid line represents the best fit adjustment of the most probable values of SS. Dashed lines represent the 95% confidence interval of the analytical extreme value distribution. (TIF).
(TIF)

S3 Fig Sensitivity Test A: TWL pre-habitat and flood height extreme distributions with and without mangroves in Tabayas Bay (Pagbilao). (A) TWL pre-habitat. (B1) Flood Height distribution produced by historical tropical cyclones (solid line) and synthetic tropical cyclones (dashed line) in case of preserving the 2010 mangrove cover. (B2) Flood Height distribution produced by historical tropical cyclones (solid line) and synthetic tropical cyclones (dashed line) in case of losing mangroves. (TIF).
(TIF)

S4 Fig Sensitivity Test B: Coastline segmentation in Pagbilao. (a) Example of 2 km spaced cross-shore profiles. (b) Example of 200 m space cross-shore profiles. Reprinted from ArcGIS Online maps under a CC BY license, with permission from Esri, original Copyright 2018 Esri (Basemaps supported by Esri, DigitalGlobe, GeoEye, Earthstar Geographics, CNES/Airbus Ds, USDA, AEX, Getmapping, Aerogrid, IGN, IGP, swisstopo, and the GIS User Community). (TIF).
(TIF)

S5 Fig Sensitivity Test C: Digital elevation model comparison in Pagbilao. (a) General view of Pagbilao bay. (b) Global SRTM 30 m resolution model. (c) MERIT DEM at 90 m resolution, obtained from SRTM by filtering out the vegetation height. (d) Local high resolution IFSAR DEM (5 m resolution). All the figures have been labeled between 0 and 20 m height. Reprinted from ArcGIS Online maps under a CC BY license, with permission from Esri, original Copyright 2018 Esri (Basemaps supported by Esri, DigitalGlobe, GeoEye, Earthstar Geographics, CNES/Airbus Ds, USDA, AEX, Getmapping, Aerogrid, IGN, IGP, swisstopo, and the GIS User Community). (TIF).
(TIF)

S6 Fig Sensitivity Test D: Flood method comparison in Pagbilao. (a) 1-in-50-year flooding in the presence of mangroves calculated with the bathtub method. (b) 1-in-50-year flooding in the presence of mangroves calculated with the RFSM-EDA model. Reprinted from ArcGIS Online maps under a CC BY license, with permission from Esri, original Copyright 2018 Esri (Basemaps supported by Esri, DigitalGlobe, GeoEye, Earthstar Geographics, CNES/Airbus Ds, USDA, AEX, Getmapping, Aerogrid, IGN, IGP, swisstopo, and the GIS User Community). (TIF).
(TIF)

S7 Fig Sensitivity Test E: Population datasets in Pagbilao and Lucena. (a) 1 km-resolution data GPW. (b) 100 m-resolution data Worldpop. Reprinted from ArcGIS Online maps under a CC BY license, with permission from Esri, original Copyright 2018 Esri (Basemaps supported by Esri, DigitalGlobe, GeoEye, Earthstar Geographics, CNES/Airbus Ds, USDA, AEX, Getmapping, Aerogrid, IGN, IGP, swisstopo, and the GIS User Community). (TIF).
(TIF)

S8 Fig Annual expected risks and benefits. (A1) Annual Expected Flooding with mangroves (light grey) and without mangroves (dark grey), calculated following the Baseline case, Benchmark case and each sensitivity test. (A2) Annual Expected Risk in terms of people affected by coastal flooding with mangroves (light grey) and without mangroves (dark grey), calculated following the Baseline case, Benchmark case and each sensitivity test. (A3) Annual Expected Risk in terms of property damaged by coastal flooding with mangroves (light grey) and without mangroves (dark grey), calculated following the Baseline case, Benchmark case and each sensitivity test. (B1) Annual Expected Flooding reduction due to the presence of mangroves, calculated following the Baseline case, Benchmark case and each sensitivity test. (B2) Annual Expected Benefits in terms of people protected by mangroves, calculated following the Baseline case, Benchmark case and each sensitivity test. (B3) Annual Expected Benefits in terms of property protected by mangroves, calculated following the Baseline case, Benchmark case and each sensitivity test. (TIF).
(TIF)

S9 Fig Cross-shore profile parameterization. Parameterized cross-shore profile typical from coral reef and mangroves regions. Where “h” values represent dater depth or topographic elevation, “B”, “L”, “W” and “D” represent horizontal distances and “m” values represent bottom slope. (TIF).
(TIF)

S1 Table Ranking table for valuing benefits, based on ERI index. A comparison of sensitivity of estimates of mangrove benefits (risk reduction) to 5 different factors and to the Baseline case. Baseline case in also ranked to show which elements improve benefits estimates (ranked above the Baseline) and which do not (ranked below the Baseline). Ranking table to prioritize the best-practice case of valuing mangrove´s protection capacity in three different ways: Flood reduction (left), people protected (mid) and total property benefits (right). (DOCX).
(DOCX)

S2 Table Ranking table for valuing flood risks in mangrove presence, based on ERI index. Ranking table to prioritize the best-practice case of valuing flood risks in presence of mangroves to 5 different factors and to the Baseline case. Baseline case in also ranked to show which elements improve risk estimates (ranked above the Baseline) and which do not (ranked below the Baseline). Ranking table to prioritize the best-practice case of valuing mangrove´s protection capacity in three different ways: Flood reduction (left), people protected (mid) and total property benefits (right). (DOCX).
(DOCX)

S3 Table Ranking table for valuing risks in mangrove absence, based on ERI index. Ranking table to prioritize the best-practice case of valuing flood risks in absence of mangroves to 5 different factors and to the Baseline case. Baseline case in also ranked to show which elements improve risk estimates (ranked above the Baseline) and which do not (ranked below the Baseline). Ranking table to prioritize the best-practice case of valuing mangrove´s protection capacity in three different ways: Flood reduction (left), people protected (mid) and total property benefits (right). (DOCX).
(DOCX)

S4 Table Error Rate Index (ERI). Error Rate Index (ERI) calculated at any intermediate case. (DOCX).
(DOCX)
