# Supplementary material for: Assessing the effects of using high-quality data and high-resolution models in valuing flood protection services of mangroves
Source: PLoS One. 2019 Aug 20;14(8):e0220941. doi: 10.1371/journal.pone.0220941 (PMC6701829; doi:10.1371/journal.pone.0220941)
Supplement: S1 File — (DOCX) [file pone.0220941.s001.docx]

## SUPPORTING FILE (S1 File)

## Sensitivity tests description

We implemented the multi-step methodology (Table 1) following the Baseline case first, and then stepwise introducing improvements at different levels: the number of tropical cyclones, coastal segmentation, DEM resolution, flood method and population resolution, consecutively, until reaching the fully high-resolution approach that provides the best-practice case typically applied at local scale (i.e. Benchmark case). This study analyzes the effect of introducing each single improvement by comparing different aspects as described next.

### Test A: Number of tropical cyclones (Historical vs synthetic)

We first compare the difference between using (1) 71-years (1945-2015) of historical tropical cyclones IBTrACS [1] versus (2) 1,000 years-long synthetic tropical cyclones created from a stochastic downscaling method based on Monte Carlo simulations [2], in the assessment of step 1 in the methodology. In the first approach we use the most complete global set of historical and real tropical cyclones available, including data from 12 different agencies (e.g. typhoon Haiyan in November 2013[3]). However, tropical cyclones are one-off site-specific events, and many places do not have enough data for a rigorous probabilistic analysis. Since synthetic tropical cyclones are randomly generated from the historical data, they preserve the essence of the original events, faithfully representing the characteristics of real tropical cyclones in the specific study area. Nevertheless, the high-computational effort of tropical cyclone models (e.g. Delft3D or ADCIRC) requires focusing only on the most important dynamics and carefully dealing with zones rarely affected, where the return periods may change with respect to highly affected areas.

The 548 historical tropical cyclones (TC) making landfall in the Philippines between 1945 and 2015 represent an average rate of 7.71 TC/year. Pagbilao had only 37 recorded events passing directly over the municipality, which is a rate of 0.52 TC/year. The shortage of historical tropical cyclones data is a relevant issue for statistical analysis of ocean climate. We use, therefore instead, 1,033 synthetic tropical cyclones datasets, 456 of them making landfall in Pagbilao.

Using synthetic storms series has three main benefits: First, it helps to reduce 95% confidence intervals of the theoretical Generalized Pareto Poisson distribution of maximum significant wave height (H_S_) and storm surge (SS) generated by tropical cyclones. These confidence bands are narrower when calculated from the synthetic series than from the historical ones. S1 Fig. illustrates the differences between the offshore significant wave height extreme distribution using historical (S1 Fig. A) versus synthetic tropical cyclones (S1 Fig. B). Idem for offshore maximum SS distribution (S2 Fig.). The 1-in-20-year H_S_ reduces the 95% confidence interval from ±6.61 m (±103% of the average expected value) to ±1.14 m (±13% of the average expected value) if using synthetic tropical cyclones. Second, the synthetic series are especially useful to estimate long return periods. For example, the 1-in-70-year H_S_ reduces the 95% confidence interval from ±12 m (±141% of the average expected value) to ±1.6 m (±15% of the average expected value) if synthetic tropical cyclones are used. Third, we can better predict the average expected value of H_S_ or SS by using synthetic tropical cyclones, avoiding underestimations derived from the scarcity of data and the lower probability of capturing more extreme events.

Waves and storm surge generated by tropical cyclones have a relevant contribution to the Total Water Level (TWL). The TWL pre-habitat is empirically estimated by linearly adding the Astronomical Tide (AT), SS and waves set-up contribution as 20% of the offshore H_S_ [4] (Eq. S1). We notice differences in the TWL pre-habitat if using historical versus synthetic tropical cyclones (S3 Fig. A). The extreme value distribution analysis shows that synthetic tropical cyclones predict higher TWL pre-habitat, up to +17.2% (+0.46 m) for 1-in-70-year storm (S3 Fig. A). Waves and storm surge propagation towards the coast interact with mangrove forests, which reduce the Flood Height (TWL along the coast). Extreme value distribution curves for both, synthetic and historical tropical cyclones and two different mangrove cover (2010 and no mangroves scenarios) are shown in S3 Fig. B1 and S3 Fig. B2 respectively.

TWL=AT+SS+0.2H_S_ (1)

In brief, the SS and the H_S_ produced by tropical cyclones is highly dependent, not only on the intensity, but also on the track variability. This fact leads to an underestimation of waves and sea levels associated to large return periods such as 1-in-70-year event (-11.6% in wave height, -25% in storm surge and -15.6% in offshore TWL) if using a short observation dataset of extreme events (i.e. only 37 historical tropical cyclones in Pagbilao).

### Test B: Coastline segmentation (2 km vs 200 m)

In our models we used cross-shore profiles parallel to the bathymetric gradient and equally spaced along the coastline to obtain the Flood Height. It is a proved efficient method to be applied globally [5,6] and nationally [7]. Existing global analysis of coastal ecosystems valuation used 2 km spaced profiles [5], but local scale studies require a higher resolution for the Flood Height. However, increasing the number of cross-shore profiles also increases the computational cost. With the aim of checking whether it is worth investing in increasing spatial discretization and, therefore, the number of coastline points with Flood Height data, we explored the sensitivity of using 2 km spaced cross-shore profiles (S4 Fig. a) versus 200 m (S4 Fig. b).

### Test C: DEM resolution (MERIT-90 m vs IFSAR-5 m)

Two different DEMs are used to assess the sensitivity of flooding to bottom topography: (1) MERIT, obtained after filtering vegetation of global SRTM (Shuttle Radar Terrain Mission) at a horizontal resolution of 9 arc-second (90 m) and vertical resolution of 1 m (S5 Fig. c), and (2) local topography data IFSAR (Interferometric Synthetic Aperture Radar) at a horizontal resolution of 5 m and a vertical accuracy of the order of centimeters [8] (S5 Fig. d).

For global scale projects we usually resort to, at best, 30 m global elevation model SRTM (S5 Fig. b). However, this study raises the use to the existing coarser horizontal MERIT data topography rather than the currently available global SRTM at 30 m because vegetated and urban areas are filtered and corrected, avoiding overestimated values of ground elevation and, consequently, reducing inundation errors [9]. Then, it is worth to use less horizontal resolution but higher vertical accuracy of the topography data.

Meanwhile, IFSAR has also been designed for surface data generation and faithfully represents ground elevation in sparsely-vegetated and non-core urban areas [10]. It was also demonstrated to be a suitable DEM for flood risk identification and mapping with insurance and governments purposes [11]. In local studies, where better topography datasets are available, the flooding simulation process might be better estimated. Among the advantages offered by IFSAR it includes (1) increased vertical accuracy, (2) higher horizontal resolution that allows us to use process-based models to simulate coastal flooding, and (3) better representation of vegetated areas [12], despite MERIT DEM already removed vegetation to avoid overestimations of ground level in forest areas (S5 Fig. d).

### Test D: Flood method (Bathtub method vs RFSM-EDA model)

Two methods were compared: the hydraulically connected “bathtub” method [5] and the process-based model RFSM-EDA [13].

The “bathtub” method consists on flooding grid cells below the water level. Hydraulic connectivity must be assured by considering that the grid cell becomes flooded if any of the 8 directions (diagonals included) are connected. This simple approach only requires the use of GIS software (Geographic Information System) and the DEM to evaluate maximum water inland reach.

The RFSM-EDA (Rapid Flood Spreading Method – Explicit Diffusion wave with Acceleration term [14,15]) is a hydraulic inundation model based on water distribution according to the floodplain topography. This process-based model admits a complete terrain information, not only the vertical elevation data but also Land Cover Maps (LCMs), in order to associate different Manning rugosity to model soil resistance. A recent version of the model RFSM-EDA uses the continuity and diffusion wave approach, including the local acceleration term and introduces the time-dependence that already outputs the maximum flooding envelopes within a storm event [14]. The complexity of this model arises from the pre-processing, which consists on producing a coarse mesh from the topography data.

The computational effort is usually the key variable to measure the efficiency of modeling methods. However, the time-required by the RFSM-EDA and bathtub does not revert on the computational time. Both models need of the order of 1 minute to simulate 1-in-50-year tropical cyclone induced flooding in Pagbilao (20 km of coastline) over a 5 m resolution DEM. The critical time requirements come from the pre-processing step. While bathtub method does not use a specific numerical mesh and directly computes flooding over the existing topography, the RFSM-EDA is very sensitive to mesh quality.

The differences between both methods to estimate the land area flooded is shown in S6 Fig., where 1-in-50-year tropical cyclone induced flooding is calculated over the high-resolution topography data (IFSAR 5 m). In the figure we notice the slightly overestimation of inland flooding produced by the bathtub method. However, flood depth is higher if using the RFSM-EDA model, which balances the damage results obtained by both methods.

### Test E: Population resolution (GWP-1 km vs WorldPop-100 m)

We compare global population data provided at two different resolutions: 1 km grid cell data [Gridded Population of the World (GPW)] (S7 Fig. a) and 100 m [WorldPop] (S7 Fig. b). Both differ in several aspects such as the origin of data, the methodologies to distribute people and the spatial resolution. This last consideration will constrain the scale at which it is worthwhile to apply one or the other. GPW could suit global projects being appropriate when dealing with large coastal areas (more than one country), while high resolution local analysis, which allows to obtain coastal impacts with detailed enough to match with high resolution socioeconomic information (e.g. 5 m horizontal grid), will likely draw on WorldPop database.

GPW is produced from census population databases using non-spatial information (e.g. tabular counts of population listed by administrative area) and spatially-explicit administrative boundary data (administrative or enumeration units). The model outputs the number of people per pixel with a spatial resolution of 1 km and different time horizons (2000, 2005, 2010, 2015, and 2020).

The WorldPop provides detailed and freely-available population distribution and composition maps for the whole of Central and South America, Africa and Asia at 10 m resolution. The production of the spatial datasets principally follows different methodologies outlined in the literature [16,17]. This involves the use of land cover data, random forest regressions, bottom-up population mapping and special intra-urban population mapping.

As well as it is feasible to obtain world population distribution data at different spatial resolutions, industrial and residential stock is no longer available at spatial resolutions finer than 5 km. Both, local and global approaches are developed by using Global Assessment Report on Disaster Risk Reduction (GAR) for the year 2015. This database provides a property value, including residential, industrial, service and government.

Adapting people distribution to the DEM resolution requires to downscale population grid from 1 km to 90 m, in case of global approaches (MERIT), and from 100 m to 5 m (IFSAR) in case of local projects. Meanwhile, property databases must be downscaled from 5 km to 90 and 5 m respectively. The abrupt downscaling process will likely result in several distribution errors, especially when dealing with 5 m grids. To minimize them, we use population distribution as a control variable. Comparing both, population and property at 90 and 5 m allows to validate the downscaled economic data distribution and identify discrepancies between GPW and WorldPop.

1. Knapp KR, Kruk MC, Levinson DH, Diamond HJ, Neumann CJ. The international best track archive for climate stewardship (IBTrACS) unifying tropical cyclone data. Bull Am Meteorol Soc. 2010;91: 363–376. doi:10.1007/978-90-481-3109-9_26

2. Nakajo S, Mori N, Yasuda T, Mase H. Global stochastic tropical cyclone model based on principal component analysis and cluster analysis. J Appl Meteorol Climatol. 2014;53: 1547–1577. doi:10.1175/JAMC-D-13-08.1

3. Mori N, Kato M, Kim S, Mase H, Shibutani Y, Takemi T, et al. Local amplification of storm surge by Super Typhoon Haiyan in Leyte Gulf. Geophys Res Lett. 2014;41: 5106–5113. doi:10.1002/2014GL060689

4. Guza RT, Thornton EB. Wave set‐up on a natural beach. J Geophys Res Ocean. 1981;86: 4133–4137.

5. Beck MW, Losada IJ, Mendendez P, Reguero BG, Díaz-Simal P, Fernandez F. The global flood protection savings provided by coral reefs. Nat Commun. 2017; doi:10.1038/s41467-018-04568-z

6. Losada IJ, Menéndez P, Espejo A, Torres S, Díaz-Simal P, Abad S, et al. The global value of mangroves for risk reduction. Technical Report. Berlin; 2018. doi:10.7291/V9DV1H2S

7. Losada IJ, Beck M, Menendez P, Espejo A, Torres S, Diaz-Simal P, et al. Valuing Protective Services of Mangroves in the Philippines. World Bank, Washington, DC; 2017.

8. Hashim S, Mohd W, Wan N. Evaluation of Vertical Accuracy of Airborne IFSAR and Open Source Digital Elevation Models ( DEMs ) Based on GPS Observation Evaluation of Vertical Accuracy of Airborne IFSAR and Open Source Digital Elevation Models ( DEMs ) Based on GPS Observation. 2016; doi:10.15242/IJCCIE.D0315014

9. Baugh CA, Bates PD, Schumann G, Trigg MA. SRTM vegetation removal and hydrodynamic modeling accuracy. 2013;49: 5276–5289. doi:10.1002/wrcr.20412

10. Mercer B, Mercer B. Comparing LIDAR and IFSAR : What can you expect ? In Proceedings of Photogrammetric Week. Stuttgart, Germany; 2001. pp. 2–10.

11. Sanders R, Shaw F, Mackay H, Galy H, Foote M, Sanders R, et al. National flood modelling for insurance purposes : using IFSAR for flood risk estimation in Europe. Hydrol Earth Syst Sci Discuss Eur Geosci Union. 2005;9: 449–456. Available: https://hal.archives-ouvertes.fr/hal-00304854%0D

12. Mohd W, Wan N, Abdullah MA, Hashim S. Evaluation of Vertical Accuracy of Digital Elevation Models Generated from Different Sources : Case Study of Ampang and Hulu Langat. FIG Congress. Malaysia; 2014. pp. 1–17.

13. Toimil A, Losada IJ, Díaz-Simal P, Izaguirre C, Camus P. Multi-sectoral , high-resolution assessment of climate change consequences of coastal flooding. 2017; 431–444. doi:10.1007/s10584-017-2104-z

14. Jamieson SR, Wright G, Lhomme J, Gouldby BP. Validation of a computationally efficient 2D inundation model on multiple scales. In: Klijn F, Schweckendiek T, editors. Comprehensive flood risk management: research for policy and practice. Rotterdam: Taylor & Francis Group; 2012. pp. 121–122.

15. Gouldby B, Lhomme J, Mcgahey C, Panzeri M, Hassan M, Burgada NK, et al. A flood system risk analysis model with dynamic sub-element 2D inundation model , dynamic breach growth and life- loss. : 1–13.

16. Stevens FR, Gaughan AE, Linard C, Tatem AJ. Disaggregating census data for population mapping using Random forests with remotely-sensed and ancillary data. PLoS One. 2015;10: 1–22. doi:10.1371/journal.pone.0107042

17. Gaughan AE, Stevens FR, Linard C, Jia P, Tatem AJ. High Resolution Population Distribution Maps for Southeast Asia in 2010 and 2015. PLoS One. 2013;8: e55882. doi:10.1371/journal.pone.0055882
