# Supplementary figures and images for: Assessing the effects of using high-quality data and high-resolution models in valuing flood protection services of mangroves
Source: PLoS One. 2019 Aug 20;14(8):e0220941. doi: 10.1371/journal.pone.0220941 (PMC6701829; doi:10.1371/journal.pone.0220941)

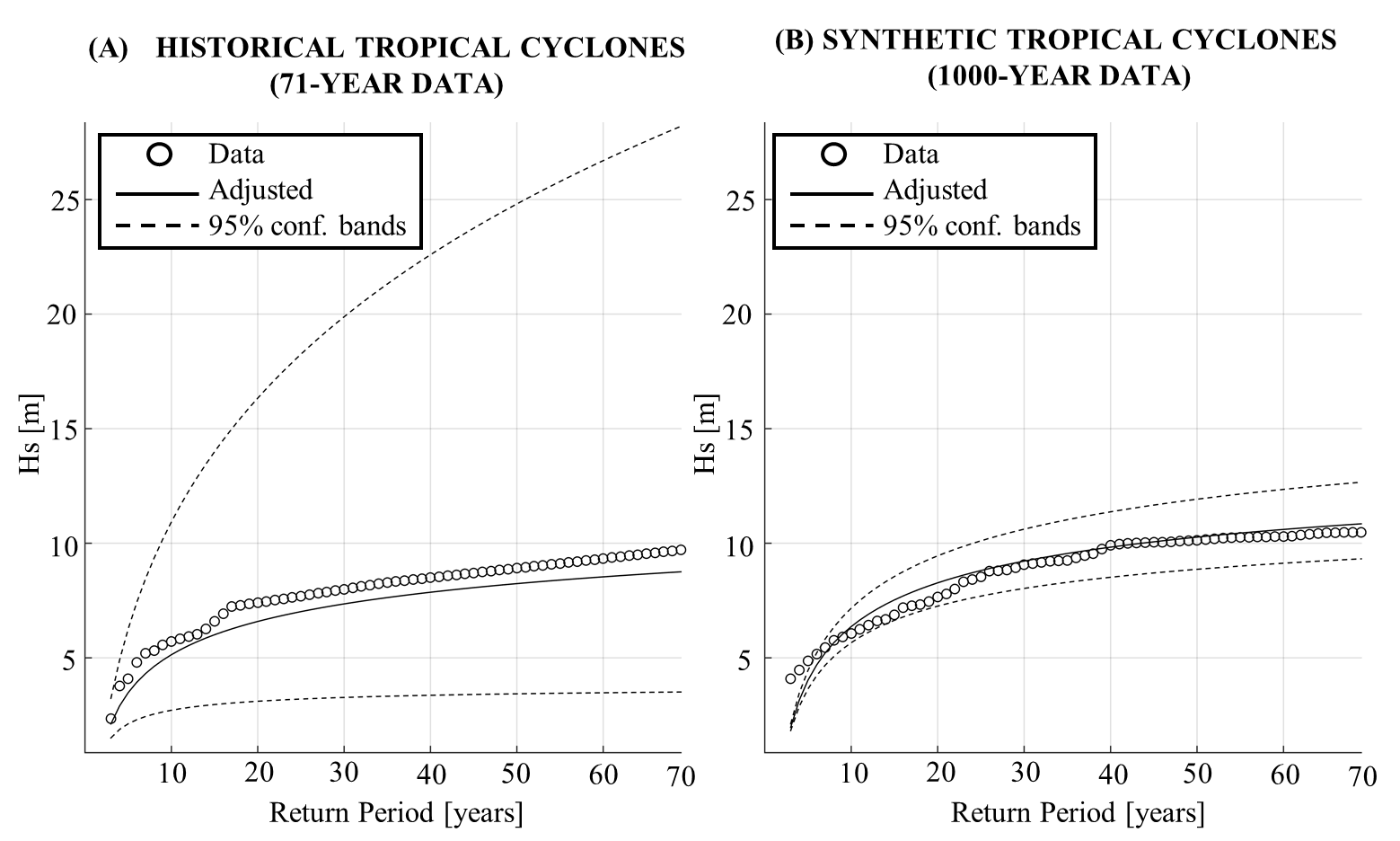

Supplement: S1 Fig — Offshore maximum significant wave height extreme distribution produced by (A) historical tropical cyclones and (B) synthetic tropical cyclones. Black circles represent the most probable value of HS. The solid line represents the best fit adjustment of the most probable values of HS. Dashed lines represent the 95% confidence interval of the analytical extreme value distribution. (TIF). (TIF) [file pone.0220941.s002.tif]

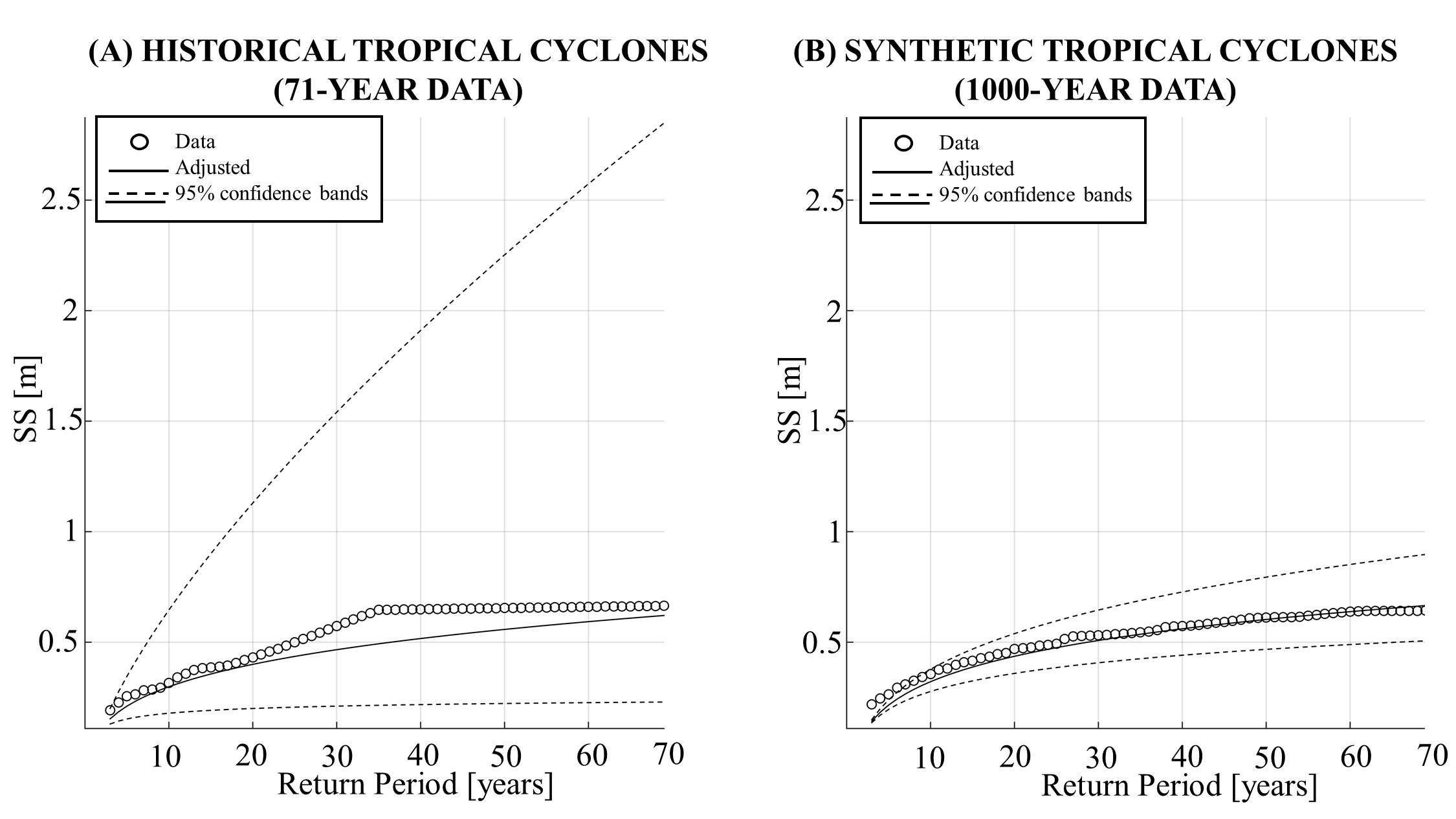

Supplement: S2 Fig — (A) Offshore maximum storm surge produced by historical tropical cyclones. (B) Offshore maximum storm surge produced by synthetic tropical cyclones. Black circles represent the most probable value of SS. The solid line represents the best fit adjustment of the most probable values of SS. Dashed lines represent the 95% confidence interval of the analytical extreme value distribution. (TIF). (TIF) [file pone.0220941.s003.tif]

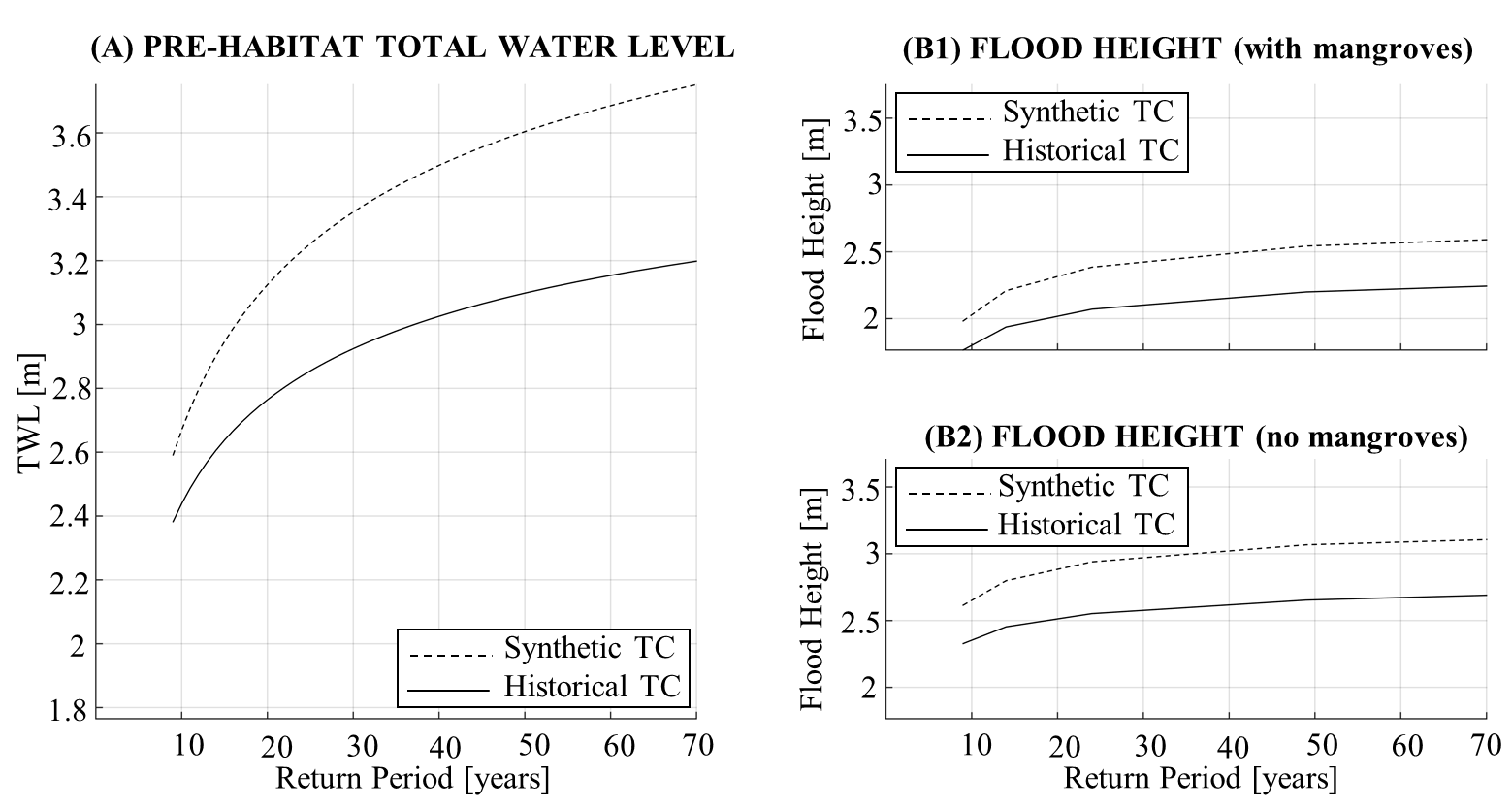

Supplement: S3 Fig — (A) TWL pre-habitat. (B1) Flood Height distribution produced by historical tropical cyclones (solid line) and synthetic tropical cyclones (dashed line) in case of preserving the 2010 mangrove cover. (B2) Flood Height distribution produced by historical tropical cyclones (solid line) and synthetic tropical cyclones (dashed line) in case of losing mangroves. (TIF). (TIF) [file pone.0220941.s004.tif]

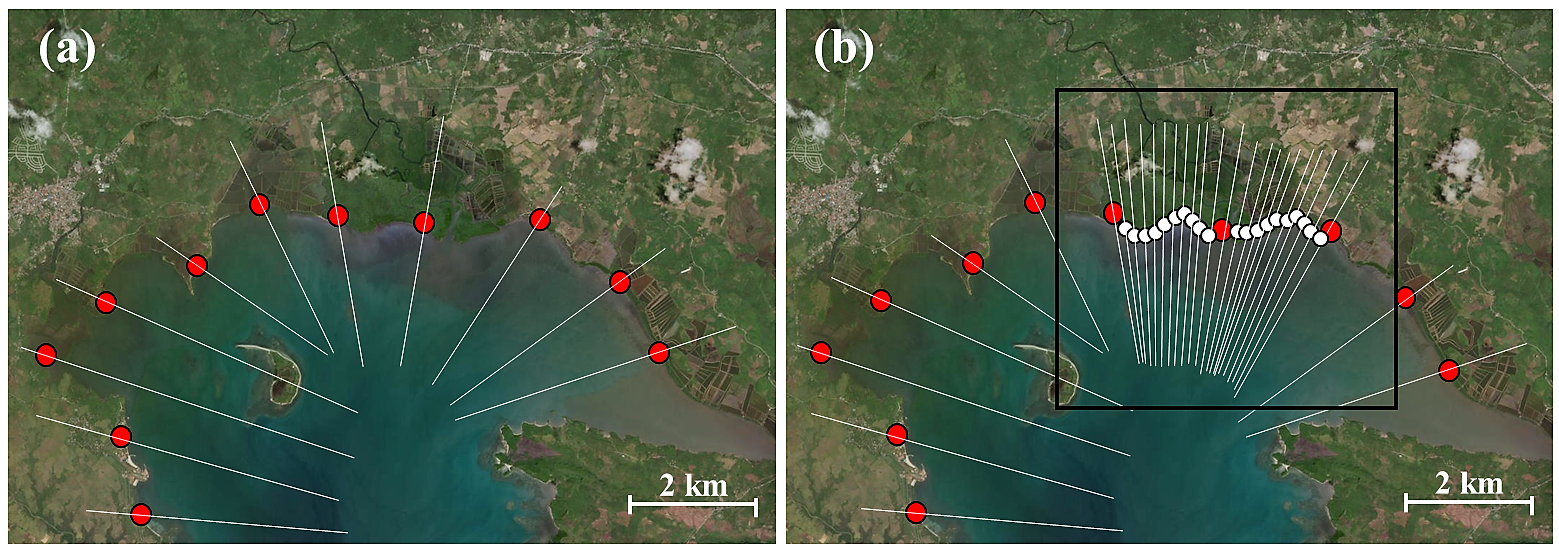

Supplement: S4 Fig — (a) Example of 2 km spaced cross-shore profiles. (b) Example of 200 m space cross-shore profiles. Reprinted from ArcGIS Online maps under a CC BY license, with permission from Esri, original Copyright 2018 Esri (Basemaps supported by Esri, DigitalGlobe, GeoEye, Earthstar Geographics, CNES/Airbus Ds, USDA, AEX, Getmapping, Aerogrid, IGN, IGP, swisstopo, and the GIS User Community). (TIF). (TIF) [file pone.0220941.s005.tif]

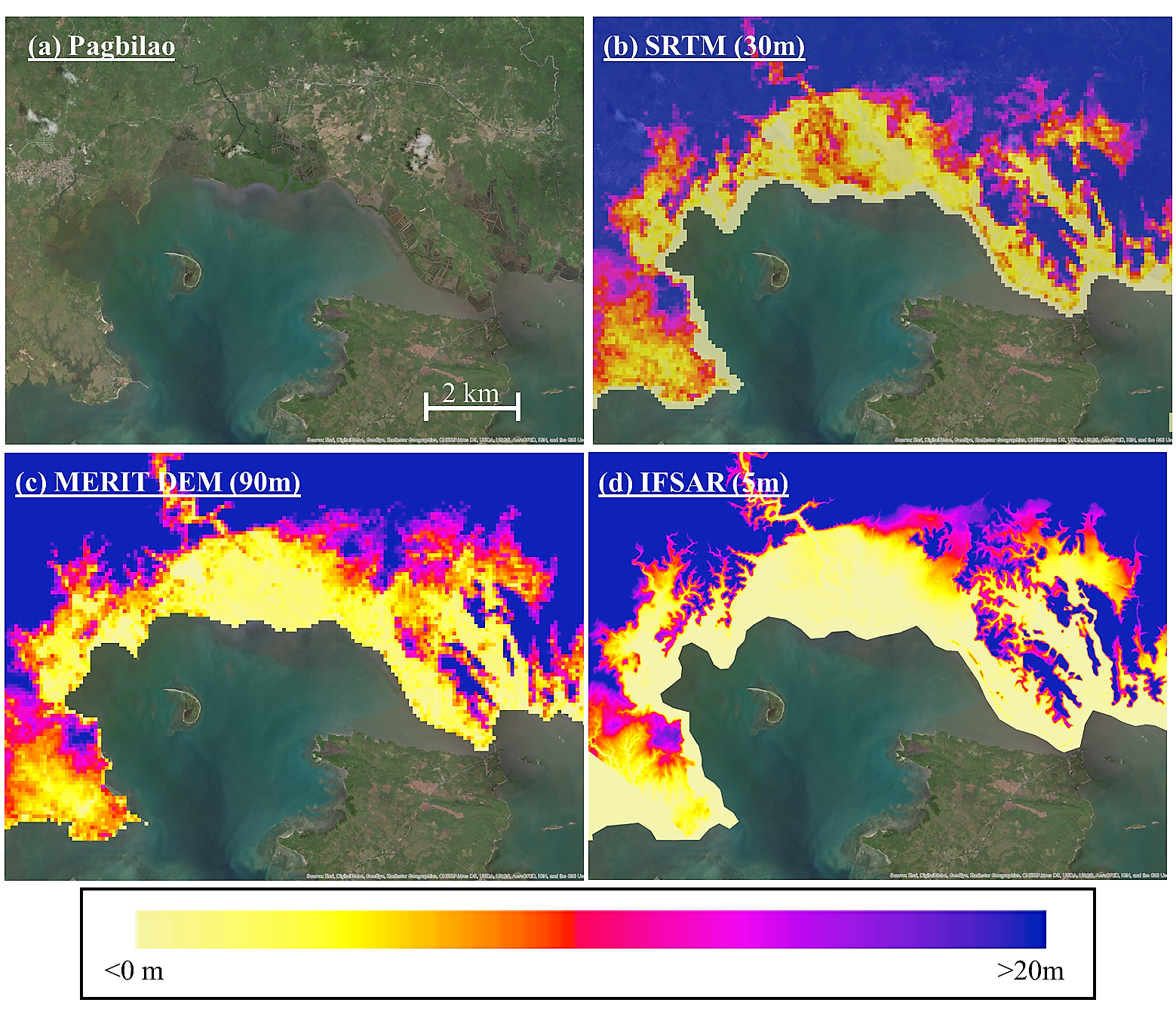

Supplement: S5 Fig — (a) General view of Pagbilao bay. (b) Global SRTM 30 m resolution model. (c) MERIT DEM at 90 m resolution, obtained from SRTM by filtering out the vegetation height. (d) Local high resolution IFSAR DEM (5 m resolution). All the figures have been labeled between 0 and 20 m height. Reprinted from ArcGIS Online maps under a CC BY license, with permission from Esri, original Copyright 2018 Esri (Basemaps supported by Esri, DigitalGlobe, GeoEye, Earthstar Geographics, CNES/Airbus Ds, USDA, AEX, Getmapping, Aerogrid, IGN, IGP, swisstopo, and the GIS User Community). (TIF). (TIF) [file pone.0220941.s006.tif]

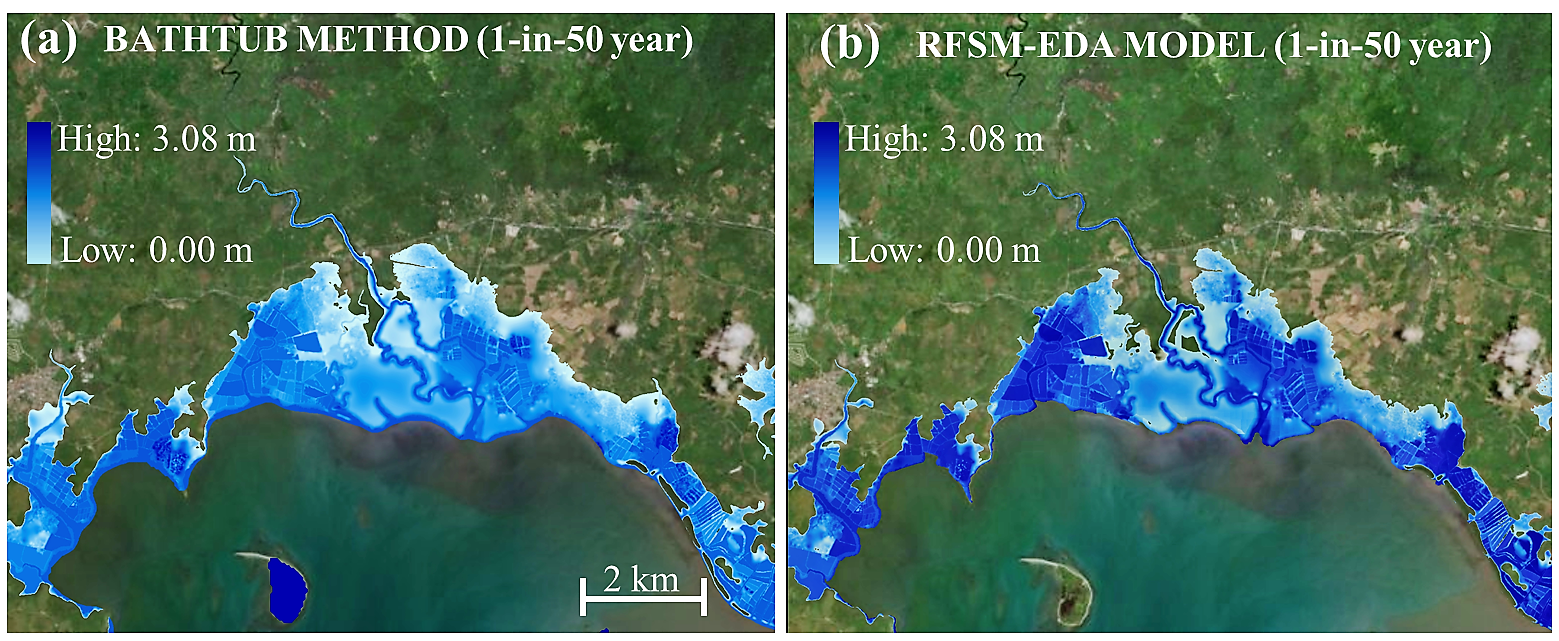

Supplement: S6 Fig — (a) 1-in-50-year flooding in the presence of mangroves calculated with the bathtub method. (b) 1-in-50-year flooding in the presence of mangroves calculated with the RFSM-EDA model. Reprinted from ArcGIS Online maps under a CC BY license, with permission from Esri, original Copyright 2018 Esri (Basemaps supported by Esri, DigitalGlobe, GeoEye, Earthstar Geographics, CNES/Airbus Ds, USDA, AEX, Getmapping, Aerogrid, IGN, IGP, swisstopo, and the GIS User Community). (TIF). (TIF) [file pone.0220941.s007.tif]

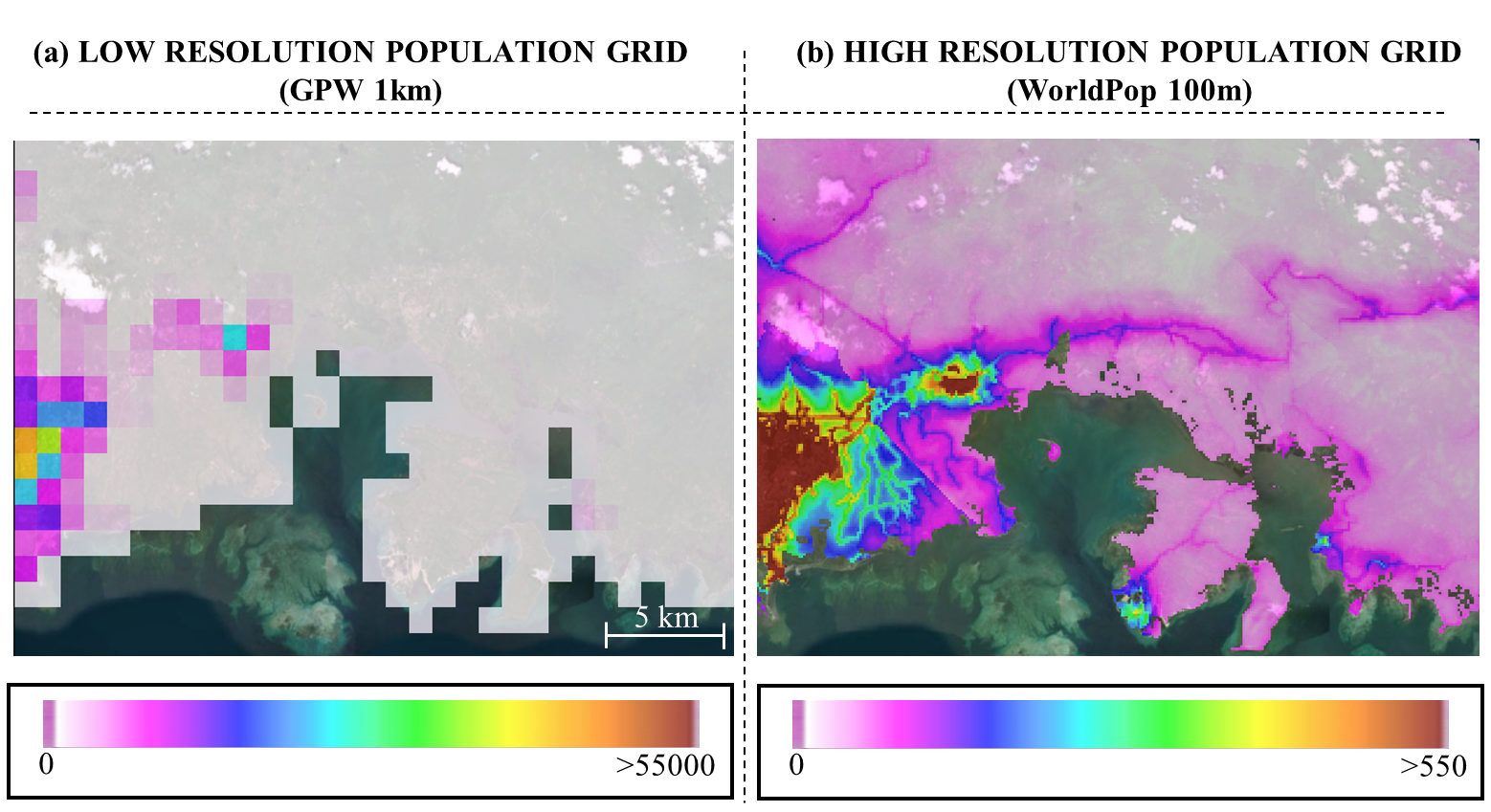

Supplement: S7 Fig — (a) 1 km-resolution data GPW. (b) 100 m-resolution data Worldpop. Reprinted from ArcGIS Online maps under a CC BY license, with permission from Esri, original Copyright 2018 Esri (Basemaps supported by Esri, DigitalGlobe, GeoEye, Earthstar Geographics, CNES/Airbus Ds, USDA, AEX, Getmapping, Aerogrid, IGN, IGP, swisstopo, and the GIS User Community). (TIF). (TIF) [file pone.0220941.s008.tif]

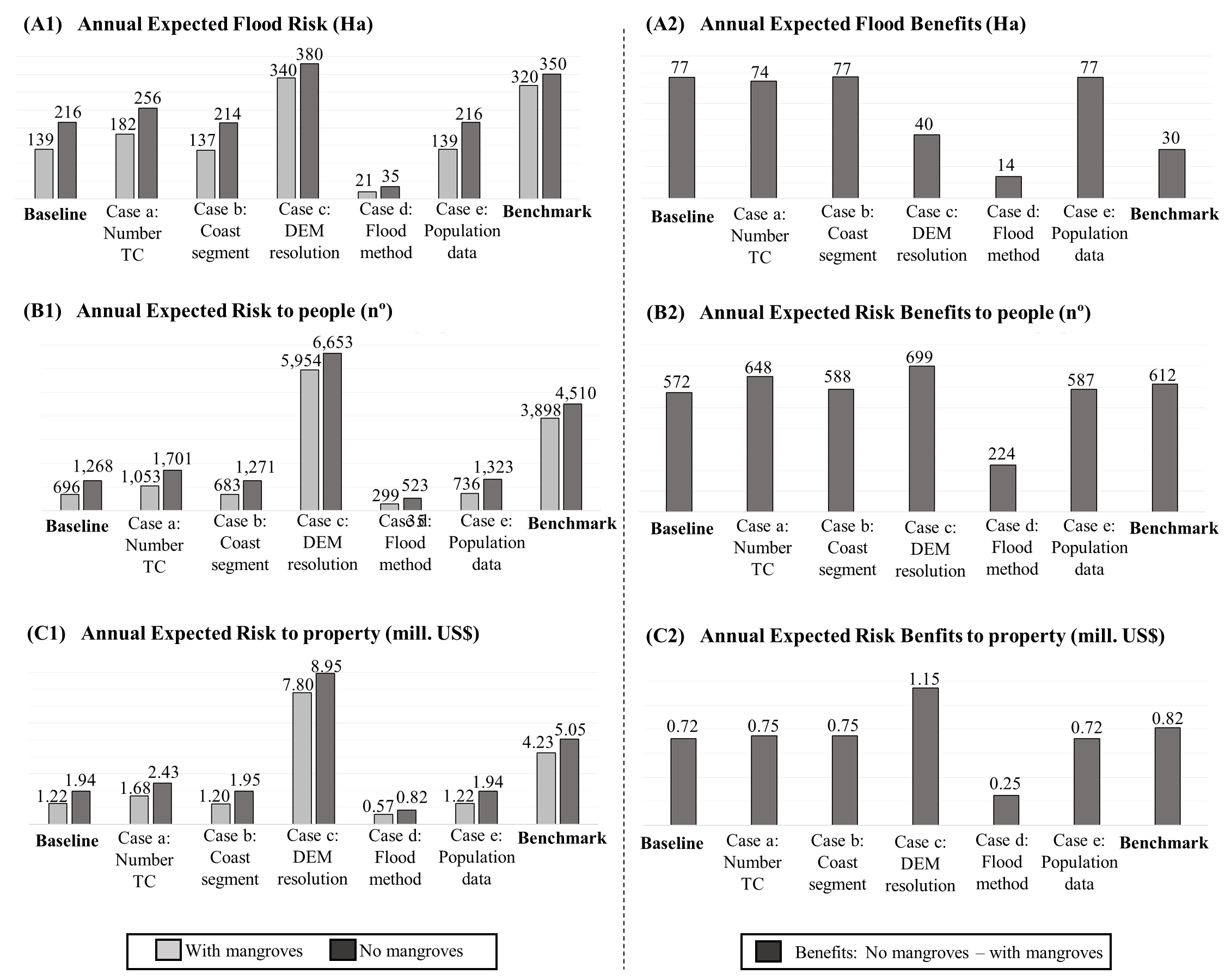

Supplement: S8 Fig — (A1) Annual Expected Flooding with mangroves (light grey) and without mangroves (dark grey), calculated following the Baseline case, Benchmark case and each sensitivity test. (A2) Annual Expected Risk in terms of people affected by coastal flooding with mangroves (light grey) and without mangroves (dark grey), calculated following the Baseline case, Benchmark case and each sensitivity test. (A3) Annual Expected Risk in terms of property damaged by coastal flooding with mangroves (light grey) and without mangroves (dark grey), calculated following the Baseline case, Benchmark case and each sensitivity test. (B1) Annual Expected Flooding reduction due to the presence of mangroves, calculated following the Baseline case, Benchmark case and each sensitivity test. (B2) Annual Expected Benefits in terms of people protected by mangroves, calculated following the Baseline case, Benchmark case and each sensitivity test. (B3) Annual Expected Benefits in terms of property protected by mangroves, calculated following the Baseline case, Benchmark case and each sensitivity test. (TIF). (TIF) [file pone.0220941.s009.tif]

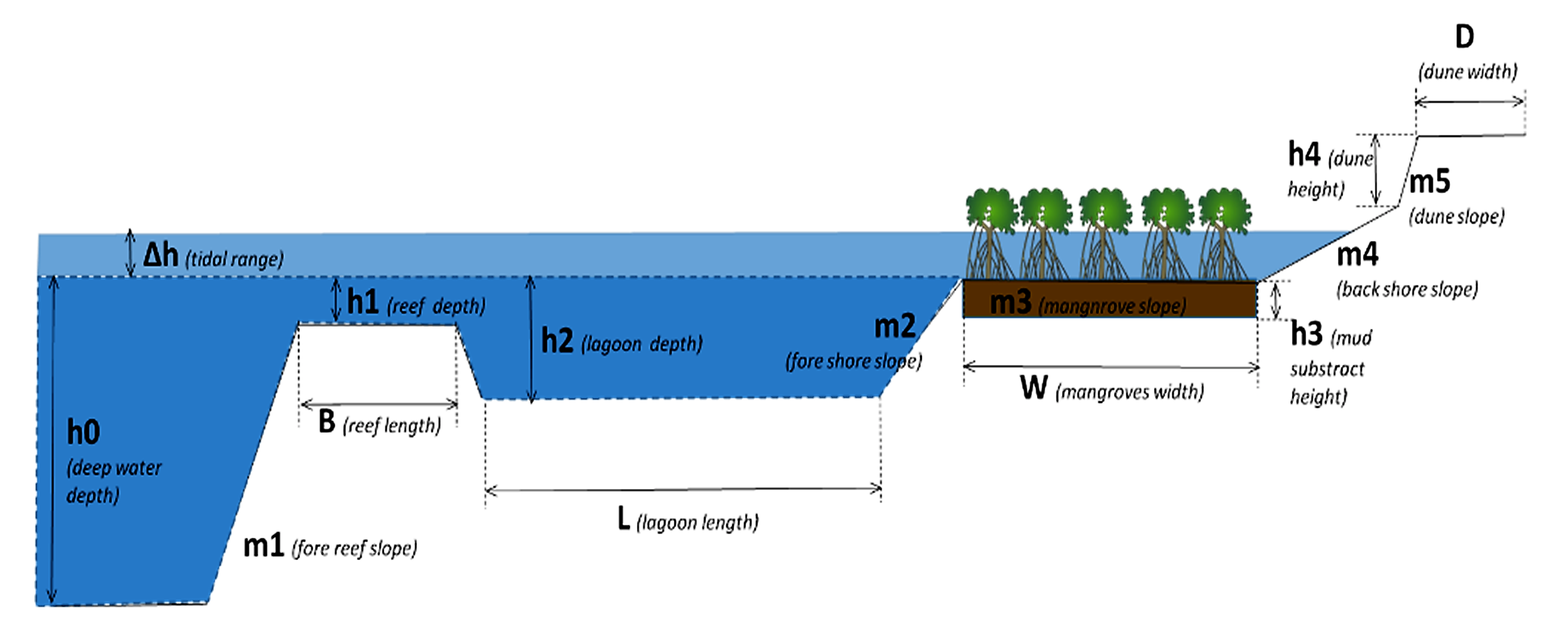

Supplement: S9 Fig — Parameterized cross-shore profile typical from coral reef and mangroves regions. Where “h” values represent dater depth or topographic elevation, “B”, “L”, “W” and “D” represent horizontal distances and “m” values represent bottom slope. (TIF). (TIF) [file pone.0220941.s010.tif]
