# Supplementary material for: Assessing the effects of using high-quality data and high-resolution models in valuing flood protection services of mangroves
Source: PLoS One. 2019 Aug 20;14(8):e0220941. doi: 10.1371/journal.pone.0220941 (PMC6701829; doi:10.1371/journal.pone.0220941)
Supplement: S1 Table — A comparison of sensitivity of estimates of mangrove benefits (risk reduction) to 5 different factors and to the Baseline case. Baseline case in also ranked to show which elements improve benefits estimates (ranked above the Baseline) and which do not (ranked below the Baseline). Ranking table to prioritize the best-practice case of valuing mangrove´s protection capacity in three different ways: Flood reduction (left), people protected (mid) and total property benefits (right). (DOCX). (DOCX) [file pone.0220941.s011.docx]

## Supporting Tables

**S1 Table. Ranking table for valuing benefits, based on ERI index**

| **BENEFITS** | | | | | | | | |
| --- | --- | --- | --- | --- | --- | --- | --- | --- |
| **LAND** | | | **PEOPLE** | | | **PROPERTY** | | |
| **Rank** | **Sensitivity test** | **ERI** | **Rank** | **Sensitivity test** | **ERI** | **Rank** | **Sensitivity test** | **ERI** |
| 1 | DEM res. | +33.33% | 1 | Nº profiles | -3.92% | 1 | Nº profiles | -8.54% |
| 2 | Flood method | -53.33% | 2 | Exposure res. (Pop) | -4.08% | 2 | Nº storms | -8.54% |
| 3 | Nº storms | +146.67% | 3 | Nº storms | +5.88% | 3 | Baseline case | -12.20% |
| 4 | Baseline case | +156.67% | 4 | Baseline case | -6.54% | 4 | Exposure res. (Pop) | -12.20% |
| 5 | Nº profiles | +156.67% | 5 | DEM res. | +14.22% | 5 | DEM res. | -40.24% |
| 6 | Exposure res. (Pop) | +156.67% | 6 | Flood method | -63.40% | 6 | Flood method | -69.51% |
