# Supplementary material for: Assessing the effects of using high-quality data and high-resolution models in valuing flood protection services of mangroves
Source: PLoS One. 2019 Aug 20;14(8):e0220941. doi: 10.1371/journal.pone.0220941 (PMC6701829; doi:10.1371/journal.pone.0220941)
Supplement: S3 Table — Ranking table to prioritize the best-practice case of valuing flood risks in absence of mangroves to 5 different factors and to the Baseline case. Baseline case in also ranked to show which elements improve risk estimates (ranked above the Baseline) and which do not (ranked below the Baseline). Ranking table to prioritize the best-practice case of valuing mangrove´s protection capacity in three different ways: Flood reduction (left), people protected (mid) and total property benefits (right). (DOCX). (DOCX) [file pone.0220941.s013.docx]

**S3 Table. Ranking table for valuing risks in mangrove absence, based on ERI index**

| **RISKS IN MANGROVES ABSENCE** | | | | | | | | |
| --- | --- | --- | --- | --- | --- | --- | --- | --- |
| **LAND FLOODED** | | | **PEOPLE AFFECTED** | | | **PROPERTY DAMAGED** | | |
| **Rank** | **Sensitivity test** | **ERI** | **Rank** | **Sensitivity test** | **ERI** | **Rank** | **Sensitivity test** | **ERI** |
| 1 | DEM res. | +8.46% | 1 | DEM res. | +47.52% | 1 | Nº storms | -51.88% |
| 2 | Nº storms | -26.86% | 2 | Nº storms | -62.28% | 2 | Nº profiles | -61.39% |
| 3 | Baseline case | -38.29% | 3 | Exposure res. (Pop) | -70.67% | 3 | Baseline case | -61.58% |
| 4 | Exposure res. (Pop) | -38.29% | 4 | Nº profiles | -71.82% | 4 | Exposure res. (Pop) | -61.58% |
| 5 | Nº profiles | -38.86% | 5 | Baseline case | -71.88% | 5 | DEM res. | +77.23% |
| 6 | Flood method | -90.00% | 6 | Flood method | -88.40% | 6 | Flood method | -83.76% |
