# Supplementary material for: Assessing the effects of using high-quality data and high-resolution models in valuing flood protection services of mangroves
Source: PLoS One. 2019 Aug 20;14(8):e0220941. doi: 10.1371/journal.pone.0220941 (PMC6701829; doi:10.1371/journal.pone.0220941)
Supplement: S4 Table — Error Rate Index (ERI) calculated at any intermediate case. (DOCX). (DOCX) [file pone.0220941.s014.docx]

**S4 Table. Error Rate Index (ERI)**

|  |  | **RISKS** | | | | | | | | | | | | | | |
| --- | --- | --- | --- | --- | --- | --- | --- | --- | --- | --- | --- | --- | --- | --- | --- | --- |
|  |  | **AVERAGE ERI** | | | **PER RETURN PERIOD** | | | | | | **MANGROVES** | | | **NO MANGROVES** | | |
|  |  | **FLOOD** | **PEOPLE** | **PROPERTY** | **ANNUAL EXPECTED** | **1-IN-10 YEAR** | **1-IN-25 YEAR** | **1-IN-50 YEAR** | **1-IN-100 YEAR** | **1-IN-200 YEAR** | **FLOOD** | **PEOPLE** | **PROPERTY** | **FLOOD** | **PEOPLE** | **PROPERTY** |
| **1** | **BASELINE CASE (GLOBAL): Hist TC+2km+MERIT+Bathtub+GWP1km-30m** | -49.16% | -81.04% | -72.16% | -53.53% | -53.53% | -58.31% | -49.82% | -46.14% | -44.80% | -62.92% | -89.09% | -83.81% | -35.31% | -71.89% | -61.50% |
| 2 | Test “a” (TC): ***Synth.TC****+2km+MERIT+Bathtub+GWP1km-30m* | -36.96% | -72.75% | -62.20% | -42.56% | -42.56% | -47.93% | -39.77% | -33.57% | -29.77% | -50.63% | -82.98% | -74.42% | -23.59% | -62.29% | -51.91% |
| 3 | Test “b” (Coast. Prof): *Hist TC+****200m****+MERIT+Bathtub+GWP1km-30m* | -49.22% | -80.92% | -71.81% | -53.80% | -53.80% | -58.37% | -50.80% | -46.61% | -44.54% | -61.42% | -88.46% | -82.46% | -35.94% | -71.83% | -61.39% |
| 4 | Test “c” (DEM): *Hist TC+2km+****IFSAR****+Bathtub+GWP1km-30m* | 6.69% | 50.98% | 81.97% | 5.91% | 5.91% | 4.93% | 6.90% | 7.16% | 7.40% | 4.14% | 52.65% | 84.29% | 8.46% | 47.51% | 77.30% |
| 5 | Test “d” (Flood Model): *Hist TC+2km+SRTM+****RFSM-EDA****+GWP1km-30m* | -91.83% | -92.04% | -86.81% | -92.86% | -92.86% | -93.76% | -92.32% | -91.66% | -90.61% | -94.50% | -95.38% | -90.14% | -88.56% | -88.40% | -83.75% |
| 6 | Test “e” (Pop.): *Hist TC+2km+SRTM+Bathtub+****WordPop100m-5m*** | -49.16% | -79.61% | -72.16% | -53.53% | -53.53% | -58.31% | -49.82% | -46.14% | -44.80% | -62.92% | -87.05% | -83.81% | -35.31% | -70.66% | -61.50% |
| 7 | Comb. 2 Tests: a & b (TCs+Coast. Prof.): ***Synth.TC****+****200m****+MERIT+Bathtub+GWP1km-30m* | -37.02% | -72.64% | -61.84% | -42.82% | -42.82% | -47.98% | -40.75% | -34.04% | -29.51% | -49.14% | -82.36% | -73.07% | -24.21% | -62.23% | -51.80% |
| 8 | Comb. 2 Tests: a & c (TCs+DEM): ***Synth.TC****+2km+****IFSAR****+Bathtub+GWP1km-30m* | 18.89% | 59.26% | 91.94% | 16.89% | 16.89% | 15.32% | 16.96% | 19.73% | 22.43% | 16.43% | 58.76% | 93.68% | 20.18% | 57.10% | 86.90% |
| 9 | Comb. 2 Tests: a & d (TCs+Flood Model): ***Synth.TC****+2km+MERIT+****RFSM-EDA****+GWP1km-30m* | -79.63% | -83.75% | -76.84% | -81.88% | -81.88% | -83.38% | -82.27% | -79.09% | -75.58% | -82.21% | -89.27% | -80.76% | -76.84% | -78.81% | -74.15% |
| 10 | Comb. 2 Tests: a & e: (TCs+Pop): ***Synth.TC****+2km+MERIT+Bathtub+****WordPop100m-5m*** | -36.96% | -71.32% | -65.19% | -42.56% | -42.56% | -47.93% | -39.77% | -33.57% | -29.77% | -50.63% | -80.94% | -74.56% | -23.59% | -61.07% | -57.54% |
| 11 | Comb. 2 Tests: b & c (Coast. Prof.+DEM): *Hist TC+****200m****+****IFSAR****+Bathtub+GWP1km-30m* | 6.63% | 51.09% | 82.32% | 5.64% | 5.64% | 4.88% | 5.92% | 6.69% | 7.67% | 5.64% | 53.28% | 85.64% | 7.83% | 47.57% | 77.41% |
| 12 | Comb. 2 Tests: b & d (Coast. Prof.+Flood Model): *Hist TC+****200m****+MERIT+****RFSM-EDA****+GWP1km-30m* | -91.89% | -91.92% | -86.46% | -93.12% | -93.12% | -93.82% | -93.30% | -92.13% | -90.35% | -93.00% | -94.75% | -88.79% | -89.19% | -88.34% | -83.64% |
| 13 | Comb. 2 Tests: b & e (Coast. Prof.+Pop.): *Hist TC+****200m****+MERIT+Bathtub+****WordPop100m-5m*** | -49.22% | -79.49% | -74.80% | -53.80% | -53.80% | -58.37% | -50.80% | -46.61% | -44.54% | -61.42% | -86.42% | -82.60% | -35.94% | -70.60% | -67.03% |
| 14 | Comb. 2 Tests: c & d (DEM+Flood Model): *Hist TC+2km+****IFSAR****+****RFSM-EDA****+GWP1km-30m* | -35.98% | 39.98% | 67.33% | -33.41% | -33.41% | -30.52% | -35.60% | -38.36% | -38.41% | -27.44% | 46.36% | 77.95% | -44.80% | 30.99% | 55.06% |
| 15 | Comb. 2 Tests: c & e (DEM+Pop.): *Hist TC+2km+****IFSAR****+Bathtub+****WordPop100m-5m*** | 6.69% | 53.80% | 78.98% | 5.91% | 5.91% | 4.93% | 6.90% | 7.16% | 7.40% | 4.14% | 54.70% | 84.15% | 8.46% | 48.73% | 71.67% |
| 16 | Comb. 2 Tests: d & e (Flood Model+Pop.): *Hist TC+2km+SRTM+****RFSM-EDA****+****WordPop100m-5m*** | -91.83% | -90.60% | -89.80% | -92.86% | -92.86% | -93.76% | -92.32% | -91.66% | -90.61% | -94.50% | -93.33% | -90.28% | -88.56% | -87.18% | -89.38% |
| 17 | Comb. 3 Tests: a, b & c (TCs+Coast. Prof.+DEM): ***Synth.TC****+****200m****+****IFSAR****+Bathtub+GWP1km-30m* | 18.83% | 59.38% | 92.29% | 16.62% | 16.62% | 15.26% | 15.98% | 19.27% | 22.70% | 17.92% | 59.38% | 95.03% | 19.56% | 57.16% | 87.01% |
| 18 | Comb. 3 Tests: a, b & d (TCs+Coast. Prof.+Flood Model): ***Synth.TC****+****200m****+MERIT+****RFSM-EDA****+GWP1km-30m* | -79.69% | -83.63% | -76.49% | -82.15% | -82.15% | -83.43% | -83.25% | -79.55% | -75.31% | -80.72% | -88.65% | -79.41% | -77.46% | -78.75% | -74.04% |
| 19 | Comb. 3 Tests: a, b & e (TCs+Coast. Prof.+Pop.): ***Synth.TC****+****200m****+MERIT+Bathtub+****WordPop100m-5m*** | -37.02% | -71.20% | -64.84% | -42.82% | -42.82% | -47.98% | -40.75% | -34.04% | -29.51% | -49.14% | -80.32% | -73.21% | -24.21% | -61.01% | -57.43% |
| 20 | Comb. 3 Tests: a, c & d (TCs+DEM+Flood Model): ***Synth.TC****+2km+****IFSAR****+****RFSM-EDA****+GWP1km-30m* | -23.78% | 48.27% | 77.29% | -22.44% | -22.44% | -20.13% | -25.54% | -25.78% | -23.38% | -15.15% | 52.47% | 87.34% | -33.07% | 40.58% | 64.65% |
| 21 | Comb. 3 Tests: a, c & e (TCs+DEM+Pop): ***Synth.TC****+2km+****IFSAR****+Bathtub+****WordPop100m-5m*** | 18.89% | 60.70% | 88.95% | 16.89% | 16.89% | 15.32% | 16.96% | 19.73% | 22.43% | 16.43% | 60. 80% | 93.54% | 20.18% | 58.33% | 81.26% |
| 22 | Comb. 3 Tests: a, d & e (TCs+Flood Model+Pop.): ***Synth.TC****+2km+MERIT+****RFSM-EDA****+****WordPop100m-5m*** | -79.63% | -82.32% | -79.84% | -81.88% | -81.88% | -83.38% | -82.27% | -79.09% | -75.58% | -82.21% | -87.23% | -80.90% | -76.84% | -77.59% | -79.78% |
| 23 | Comb. 3 Tests: b, c & d (Coast. Prof.+DEM+Flood Model): *Hist TC+****200m****+****IFSAR****+****RFSM-EDA****+GWP1km-30m* | -36.04% | 40.10% | 67.68% | -33.68% | -33.68% | -30.57% | -36.58% | -38.83% | -38.14% | -25.94% | 46.99% | 79.30% | -45.42% | 31.05% | 55.17% |
| 24 | Comb. 3 Tests: b, c & e (Coast. Prof.+DEM+Pop.): *Hist TC+****200m****+****IFSAR****+Bathtub+****WordPop100m-5m*** | 6.63% | 52.53% | 79.33% | 5.64% | 5.64% | 4.88% | 5.92% | 6.69% | 7.67% | 5.64% | 55.32% | 85.50% | 7.83% | 48.79% | 71.78% |
| 25 | Comb. 3 Tests: b, d & e (Coast. Prof.+Flood Model+Pop.): *Hist TC+****200m****+MERIT+****RFSM-EDA****+****WordPop100m-5m*** | -91.89% | -90.49% | -89.45% | -93.12% | -93.12% | -93.82% | -93.30% | -92.13% | -90.35% | -93.00% | -92.71% | -88.93% | -89.19% | -87.12% | -89.27% |
| 26 | Comb. 3 Tests: c, d & e (DEM+Flood Model+Pop.): *Hist TC+2km+****IFSAR****+****RFSM-EDA****+****WordPop100m-5m*** | -35.98% | 41.41% | 64.33% | -33.41% | -33.41% | -30.52% | -35.60% | -38.36% | -38.41% | -27.44% | 48.41% | 77.81% | -44.80% | 32.21% | 49.42% |
| 27 | Comb. 4 Tests: a, b, c & d (TCs+Coast. Prof.+DEM+Flood Model): ***Synth.TC****+****200m****+****IFSAR****+****RFSM-EDA****+GWP1km-30m* | -23.84% | 48.39% | 77.64% | -22.70% | -22.70% | -20.19% | -26.52% | -26.25% | -23.11% | -13.66% | 53.09% | 88.69% | -33.70% | 40.64% | 64.77% |
| 28 | Comb. 4 Tests: a, b, c & e (TCs+Coast. Prof.+DEM+Pop.): ***Synth.TC****+****200m****+****IFSAR****+Bathtub+****WordPop100m-5m*** | 18.83% | 60.81% | 89.30% | 16.62% | 16.62% | 15.26% | 15.98% | 19.27% | 22.70% | 17.92% | 61.43% | 94.89% | 19.56% | 58.39% | 81.38% |
| 29 | Comb. 4 Tests: a, b, d & e (TCs+Coast. Prof.+Flood Model+Pop.): ***Synth.TC****+****200m****+MERIT+****RFSM-EDA****+****WordPop100m-5m*** | -79.69% | -82.20% | -79.49% | -82.15% | -82.15% | -83.43% | -83.25% | -79.55% | -75.31% | -80.72% | -86.61% | -79.54% | -77.46% | -77.53% | -79.67% |
| 30 | Comb. 4 Tests: a, c, d & e (TCs+DEM+Flood Model+Pop.): ***Synth.TC****+2km+****IFSAR****+****RFSM-EDA****+****WordPop100m-5m*** | -23.78% | 49.70% | 74.30% | -22.44% | -22.44% | -20.13% | -25.54% | -25.78% | -23.38% | -15.15% | 54.51% | 87.20% | -33.07% | 41.81% | 59.02% |
| 31 | Comb. 4 Tests: b, c, d & e (Coast. Prof.+DEM+Flood Model+Pop.): *Hist TC+****200m****+****IFSAR****+****RFSM-EDA****+WordPop100m-5m* | -36.04% | 41.53% | 64.68% | -33.68% | -33.68% | -30.57% | -36.58% | -38.83% | -38.14% | -25.94% | 49.03% | 79.16% | -45.42% | 32.27% | 49.54% |
| 32 | **BENCHMARK CASE (LOCAL HIGH RESOLUTION): Synth. TC+ 200m+IFSAR+RFSM-EDA+WordPop100m-5m** | 0% | 0% | 0% | 0% | 0% | 0% | 0% | 0% | 0% | 0% | 0% | 0% | 0% | 0% | 0% |
